# Multifunctional Tacrine–Quinoline Hybrids as Cholinesterase Inhibitors, Aβ Aggregation Blockers, and Metal Chelators for Alzheimer’s Therapy

**DOI:** 10.3390/molecules30173489

**Published:** 2025-08-25

**Authors:** Xiaohua Wang, Minglan Ma, Yalan Feng, Jian Liu, Gang Wang

**Affiliations:** 1 Synergy Innovation Center of Biological Peptide Antidiabetics of Hubei Province, College of Life Science, Wuchang University of Technology, Wuhan 430223, China; msnhm123@gmail.com (X.W.);; 2 School of Chemistry and Environmental Engineering, Wuhan Institute of Technology, Wuhan 430025, China; 22409010072@stu.wit.edu.cn

**Keywords:** acetylcholinesterase, butyrylcholinesterase, metal chelation, multitarget-directed ligands, Alzheimer’s disease

## Abstract

A novel series of multifunctional tacrine–quinoline hybrids were designed, synthesized, and evaluated as potential anti-Alzheimer’s agents. These compounds incorporate tacrine for cholinesterase’s inhibition and 8-hydroxyquinoline for metal chelation. Piperazine was selected as a linker to provide conformational flexibility and to create favorable cation–π interactions with residues in the mid-gorge region of AChE, enhancing dual-site binding with AChE to inhibit Aβ aggregation. In vitro studies demonstrated submicromolar inhibitory activity toward both AChE and BuChE, particularly for compounds **16e** (IC_50_ = 0.10 μM for AChE, 0.043 μM for BuChE) and **16h** (IC_50_ = 0.21 μM for AChE, 0.10 μM for BuChE). These compounds also exhibited potent inhibition of self-induced Aβ_1–42_ aggregation (**16e**: 80.5% ± 4.4%, 16h: 93.2% ± 3.9% at 20 μM). Kinetic analyses revealed mixed-type inhibition, suggesting dual binding to both CAS and PAS of AChE. UV–vis spectrometry confirmed the chelation of Cu^2+^ and Zn^2+^ ions by the 8-hydroxyquinoline moiety. These findings highlight the tacrine–quinoline scaffold as a promising platform for the discovery of a multitarget-directed anti-AD drug.

## 1. Introduction

Alzheimer’s disease (AD) is a well-known form of dementia, affecting an increasing number of people around the world [1]. It is an illness of the central nervous system (CNS), being a form of neurodegeneration irreversible to date, which progressively damages patients’ memory and cognition, commonly in the geriatric population [2,3,4,5,6,7]. While AD primarily affects individuals aged 65 and over, its incidence in younger populations has been increasing [8]. Currently, dementia is among the top ten causes of death worldwide, with AD accounting for 50–60% of cases. According to the 2021 report by Alzheimer’s Disease International (ADI), a new case of AD is diagnosed globally every 3.2 s [9]. Although the exact etiology of AD is still not completely known, several factors, including hyperphosphorylation of tau-protein [10], oxidative stress [11], deposits of amyloid-β (Aβ) [12,13], dyshomeostasis of biometals [14], and deficit of acetylcholine (ACh) [15], seem to play important roles in the pathophysiology of the disease. The main strategy for the treatment of AD in the past decades is to evaluate the level of ACh based on the cholinergic hypothesis [16]. This hypothesis proposed that the selective loss of cholinergic neurons in AD leads to a reduction in acetylcholine (ACh) in certain areas of the brain responsible for learning and memory. ACh is stored in synaptic vesicles for a short period. When nerve signals are received, it is released into the synaptic space and attaches to cholinergic receptors, facilitating the transmission of cholinergic nerve signals between neurons. Once the signaling is finished, ACh in the synaptic cleft is taken up by AChE and then broken down through hydrolysis [17,18,19]. In pathological conditions, the activity of AChE is increased, leading to a lower concentration of ACh in the synaptic cleft, which in turn hinders the transmission of cholinergic signals between neurons. Inhibiting the activity of AChE to prevent ACh degradation in synapses is the most important approach in medicinal chemistry, making AChE a crucial target for managing AD [20,21,22,23]. What is more, butyrylcholinesterase (BuChE) also plays a critical role in neural signaling. With its broad substrate specificity enabling the metabolism of diverse cholinergic compounds, BuChE offers compensatory metabolic support in case of an AChE deficiency, synergistically maintaining the dynamic balance of the cholinergic system [24]. This functional synergy is counterbalanced by BuChE’s dual role in neurodegeneration: it exacerbates disease’s progression through the processing of neurotoxic metabolites and neuroinflammatory modulation, highlighting its therapeutic relevance in AD’s pathology.

Based on the cholinergic hypothesis, four acetylcholinesterase (AChE) inhibitors (tacrine, donepezil, rivastigmine, and galanthamine) have been approved for commercial use by the US Food and Drug Administration (FDA). They can significantly improve the cognitive function of patients but cannot prevent the progression of dementia or cure AD [25]. Notably, tacrine has been withdrawn from clinical use because of its serious liver toxicity and related safety issues. However, various research teams are still working on it to identify effective AChE inhibitors that have minimal toxicity [26,27].

The amyloid hypothesis suggests that accumulation of Aβ peptide aggregates in the brain is thought to play an important role in the pathogenesis of AD, as their accumulation may result in a cascade of biochemical events leading to neuronal dysfunction. Therefore, the prevention of Aβ_1–42_ aggregation attracts much current attention. Crystallographic studies of AChE demonstrated two distinct binding sites. The primary site is located at the base of a deep and narrow gorge measuring 20 Å, which includes the AChE catalytic triad and an anionic subsite referred to as the catalytic active site (CAS). Additionally, there exists the peripheral anionic site (PAS) located near the entrance of the gorge [28]. The PAS plays a pivotal role, as it is believed to aid the aggregation of amyloid beta (Aβ), which contributes to the complex molecular processes linked to AD [29]. The fact that AChE accelerates Aβ aggregation, and that this effect is sensitive to PAS blockers, has led to the development of dual inhibitors of both CAS and PAS. These compounds have the potential to both enhance cognitive function and reduce the speed of Aβ aggregation, making them promising candidates for disease-modifying Alzheimer’s treatments [30,31,32].

Recent evidence has indicated that dyshomeostasis of biometals (Fe, Cu, Zn) in the brain might contribute to AD’s pathogenesis. Research has also revealed that metal ion levels in AD patients are three to seven times higher than those in healthy individuals [33]. The accumulation of Aβ can be accelerated by Cu^II^ and Zn^II^, thereby resulting mainly in the formation of amorphous aggregates, while Fe^III^ induces the formation of fibrillar Aβ plaques [34]. Additionally, transition metal ions within Cu– and Fe–amyloid complexes can be readily reduced by natural reducing agents, and the corresponding reduced forms lead to the production of reactive oxygen species (ROS) via the reduction of dioxygen. Furthermore, the Cu^II^–amyloid beta complex is implicated in mitochondrial dysfunction [35]. Thus, based on widespread evidence that high concentrations of metal ions in the senile plaques can promote Aβ aggregation and also lead to Aβ-mediated oxidative damage, the disruption of metal–Aβ peptide interaction via metal chelation has recently been used as a new therapeutic strategy against AD. The development of chelators for potential AD drugs also attracts much attention [36,37,38].

The multifactorial pathological nature and complex network of AD is believed to be the main reason for the lack of an effective drug to combat AD. Therefore, the multitarget-directed ligand (MTDL) strategy, which is a single chemical entity that is able to target two or more AD-related targets, has gained more and more attention. And encouraging results have been reported by several research groups [39,40,41]. Realizing that 8-hydroxyquinoline and its analogs, such as clioquinol and PBT2, exhibit excellent metal-chelating properties and Aβ aggregation modulation, efforts have been made to develop multifunctional hybrid molecules that combine metal-binding capacity with cholinesterase inhibition [42]. Tacrine, although withdrawn due to hepatotoxicity, remains a valuable pharmacophore for AChE inhibition, particularly when appropriately modified to reduce toxicity. Recent studies have shown that dual-binding AChE inhibitors targeting both the CAS and PAS can not only enhance cognitive function by inhibiting ACh hydrolysis but also prevent AChE-induced Aβ aggregation via PAS blockade.

Inspired by this multitarget strategy, we created and synthesized a novel series of tacrine–quinoline hybrids linked via piperazine. Designed to serve as multitarget-directed ligands for treating Alzheimer’s disease, these hybrids integrate (i) a tacrine moiety for cholinesterase’s inhibition, (ii) an 8-hydroxyquinoline or 8-aminoquinoline fragment for metal chelation, and (iii) a flexible linker to span both the CAS and PAS of AChE to prevent AChE-induced Aβ aggregation. In this work, we report the synthesis and biological evaluation of these hybrids as multitarget-directed ligands (MTDLs) for the treatment of AD. Their cholinesterase inhibitory potency, Aβ aggregation suppression, and metal-chelating capacity were systematically investigated in vitro to assess their therapeutic potential.

## 2. Result and Discussion

### 2.1. Design

**Figure 1:**
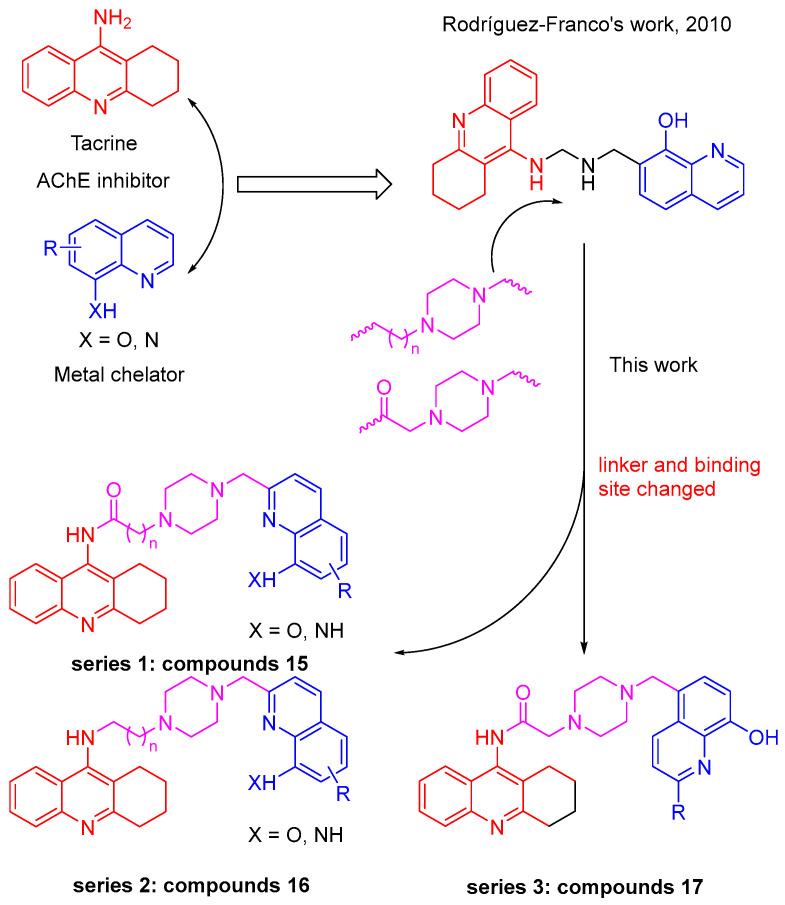
Rational design of multitarget tacrine-quinoline hybrids targeting AChE, Aβ aggregation, and metal ions for Alzheimer’s disease [43].

Tacrine displays strong binding affinity for the catalytic active site (CAS) of acetylcholinesterase (AChE) and therefore represents a potent cholinesterase pharmacophore. However, its clinical utility has been severely limited by dose-dependent hepatotoxicity, which has been mechanistically linked to the 9-amino substituent and metabolic activation of the acridine ring to reactive quinone–imine intermediates. To retain the desirable cholinesterase inhibitory potency of tacrine while mitigating its metabolic liability, we sought to modify the tacrine scaffold by conjugating it with additional functional fragments that broaden its pharmacological activity and potentially reduce hepatotoxic risk. To develop dual-site AChE inhibitors capable of simultaneously targeting both the CAS and the peripheral anionic site (PAS), we selected 8-hydroxyquinoline or 8-aminoquinoline derivatives as metal-chelating moieties, given their additional potential to modulate Aβ aggregation. These groups were tethered to the tacrine core via flexible linkers, allowing the hybrids to span the AChE gorge and simultaneously engage multiple binding domains, while providing antioxidant and metal chelation properties [43]. Recent studies have also highlighted the importance of the mid-gorge region in AChE, where aromatic residues enable cation–π interactions with protonated nitrogen atoms. To exploit this interaction and further enhance AChE’s inhibition, we introduced a piperazine ring as the linker. Piperazine not only provides conformational flexibility but also contains protonated nitrogen atoms capable of forming favorable cation–π interactions with residues within the mid-gorge region, thereby strengthening their binding affinity [44,45]. Based on this design rationale, a series of piperazine-bridged tacrine–quinoline hybrids (compounds **15**–**17**) were constructed (Figure 1). Tacrine serves as the core AChE pharmacophore, engaging the CAS and contributing to BuChE inhibition. 8-Hydroxy/aminoquinoline acts as a PAS-binding moiety and a metal-chelating group to mitigate Aβ self-aggregation and metal-induced oxidative stress. Piperazine serves as a linker to span the gorge and potentially interact with mid-gorge residues, thereby enhancing the overall binding affinity and multifunctionality.

### 2.2. Synthetic Methodology

**Scheme 1:**
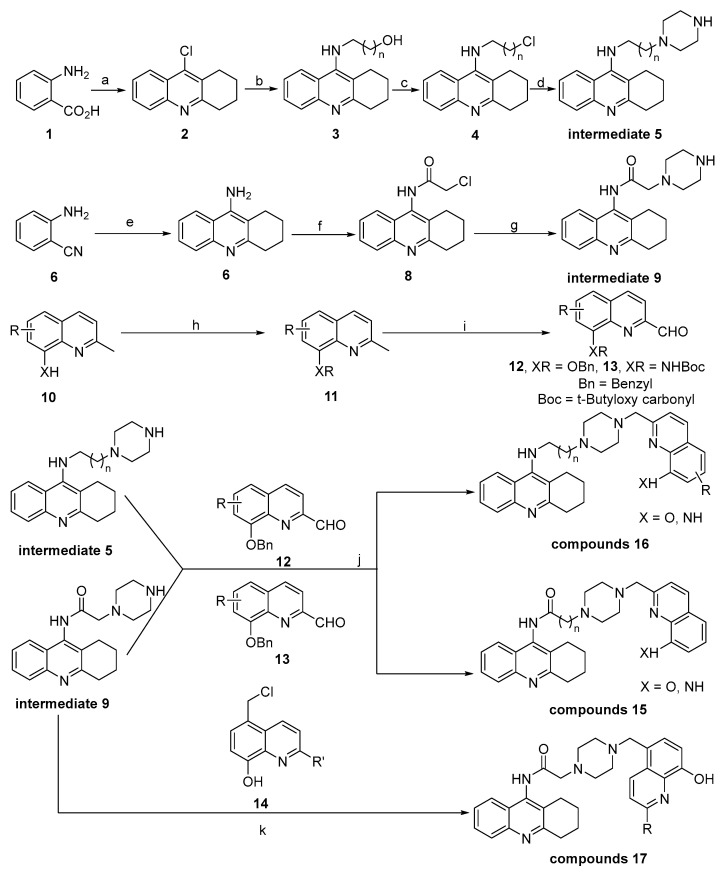
Synthesis of triazole-linked tacrine–quinoline hybrids **15**, **16**, **17**. Reagents and conditions: (a) cyclohexanone, POCl_3_, reflux, 3 h, 89%; (b) hydramine, PhOH, 130 °C, 12 h, 70–75%; (c) SOCl_2_, reflux, 4 h, 89–95%; (d) piperazine, NaI, EtOH, reflux, 4 h, 71–83%; (e) cyclohexanone, BF_3_•Et_2_O, toluene, reflux, 12 h; then KOH (50%), dioxane, reflux, 3 h, 83%; (f) chloroacetyl chloride, Et_3_N, reflux, 5 h, 87%; (g) piperazine, NaI, EtOH, reflux, 3 h, 79%; (h) BnCl, K_2_CO_3_, DMF, 80 °C, 2 h, 93% or (Boc)_2_O, dioxane, overnight, 95%; (i) SeO_2_, dioxane, 80 °C, 12 h, 81%; (j) NaBH(OAc)_3_, dichloroethane, 50 °C, 3 h; then HCl (37%), reflux, 1 h or trifluoroacetic acid, rt, 1 h; (k) K_2_CO_3_, dichloromethane, 1 h.

The piperazine-linked tacrine–8-hydroxy(amino)quinoline hybrids (**15**, **16**, **17**) were synthesized as shown in Scheme 1. First, key intermediates **5** and **9** were synthesized from 2-amino benzoic acid and 2-amino benzonitrile, respectively. 2-amino benzoic acid **1** was treated with cyclohexanone in POCl_3_, and the mixture was heated to reflux to afford 9-chloro-1,2,3,4-tetrahydroacridine **2**, then condensed with hydramines to provide compound **3**, and **3** was refluxed in SOCl_2_ to make the substitution of hydroxyl group to chloro atom, affording compound **4**, then linked with piperazine via a nucleophilic substitution to give intermediate **5**. On the other hand, 2-amino benzonitrile **6** was treated with cyclohexanone and BF_3_·Et_2_O in refluxed toluene to afford a Schiff base, then refluxed in concentrated potassium hydroxide to provide tacrine **7**; after acylation by 2-chloroacetyl chloride, compound **8** was obtained, and then piperazine was linked to the molecule by elimination of a molecule of HCl, affording another key intermediate **9**.

2-formyl quinoline derivatives **12** and **13** were synthesized from starting material **10** over two steps; first the hydroxy or amino group was protected by BnCl or Boc_2_O to obtain compound **2**, then **2** was oxidated by SeO_2_ to give key intermediate **3**. The targeted molecules **15** and **16** could be synthesized from intermediate **9** and intermediate **12** or **13** through reductive amination and deprotection. And targeted molecule **17** could be obtained by linking intermediate **9** and compound **14**, which can be synthesized from an 8-hydroxyquinoline derivative using Blanc’s chloromethylation reaction [46].

### 2.3. Cholinesterase Inhibitory Activity

The inhibitory activities of the synthesized tacrine–quinoline hybrids (compounds **15**–**17**) toward acetylcholinesterase (AChE, from an electric eel) and butyrylcholinesterase (BuChE, from horse serum) were evaluated using Ellman’s spectrophotometric method [47], with tacrine as the reference inhibitor. The IC_50_ values and selectivity indices (SI = IC_50_(AChE)/IC_50_(BuChE)) are summarized in Table 1. All compounds exhibited dual inhibitory activity against AChE and BuChE in the submicromolar to low micromolar range, indicating the general success of the hybrid design. Notably, compounds from the **16** series, in which the amide moiety of the tacrine–quinoline hybrids was replaced by a methylene linker, showed marked improvement in potency relative to the corresponding **15** series. For example, compound **15a** (IC_50_ = 2.02 μM for AChE) became **16a** (IC_50_ = 0.44 μM), and **15d** (IC_50_ = 1.18 μM) became **16b** (IC_50_ = 0.19 μM), suggesting that deacylation enhances binding affinity, likely due to increased conformational flexibility or better spatial accommodation within the AChE gorge. The length of the linker also significantly influenced the activity. When the number of methylene units (*n*) in the linker increased from **1** to **2**, the potency improved for most compounds (e.g., **16a** to **16e**, IC_50_ from 0.44 μM to 0.10 μM). However, a further extension to *n* = 4 (e.g., **16h**, IC_50_ = 0.21 μM) resulted in a moderate decrease in activity, indicating an optimal linker length at *n* = 2, possibly allowing simultaneous engagement with both the catalytic active site (CAS) and peripheral anionic site (PAS) of AChE. The nature and position of substituents on the quinoline ring also modulated the activity. Among the 8-hydroxyquinoline derivatives, 5-fluoro substitution (e.g., **16f**, IC_50_ = 0.087 μM for AChE, 0.028 μM for BuChE) led to the most potent BuChE inhibition and improved AChE inhibition compared to the unsubstituted analog (**16e**), suggesting favorable electronic effects. In contrast, 5,7-dichloro substitution (e.g., **16c**, IC_50_ = 2.68 μM) markedly reduced activity, possibly due to steric hindrance or an altered electronic distribution interfering with PAS binding.

Interestingly, replacing the 8-hydroxyquinoline with 8-aminoquinoline in the same scaffold generally enhanced inhibitory potency, as exemplified by **16g** (IC_50_ = 0.052 μM for AChE, 0.018 μM for BuChE, SI = 2.89), the most potent compound across the entire series. This suggests that the 8-amino group may better interact with PAS residues or form additional hydrogen bonds. Comparison between 2-position and 5-position quinoline substitution revealed that moving the linker from position 2 to 5 (**17a**/**17b**) significantly reduced activity (e.g., **17a**, IC_50_ = 4.46 μM for AChE), likely due to an unfavorable spatial orientation that weakens dual-site engagement. The selectivity index (SI) further supports the SAR findings. Several hybrids showed balanced or BuChE-biased profiles (e.g., **16f**, SI = 3.11; **16k**, SI = 3.89), whereas tacrine was AChE-selective (SI = 5.86). This BuChE-directed shift could be beneficial in late-stage AD, where BuChE progressively compensates for AChE deficiency.

In summary, the most potent and well-balanced dual ChE inhibitors were found among the **16** series, particularly compounds **16e**, **16f**, **16g**, and **16h**, with IC_50_ values in the low nanomolar range for both enzymes. These results validate the design rationale and highlight the importance of linker length, quinoline substitution, and amide removal in modulating dual-site inhibition.

### 2.4. Kinetic Study of AChE Inhibition

To elucidate the mechanism of AChE’s inhibition by the synthesized hybrids, a detailed enzyme kinetic study was conducted using electric eel AChE (eeAChE). Compound **16e** was selected as a representative candidate. The enzyme activity was measured at varying concentrations of the substrate acetylthiocholine (0.05–1.0 μM) in the absence and presence of increasing concentrations of **16e**, using Ellman’s spectrophotometric method.

**Figure 2:**
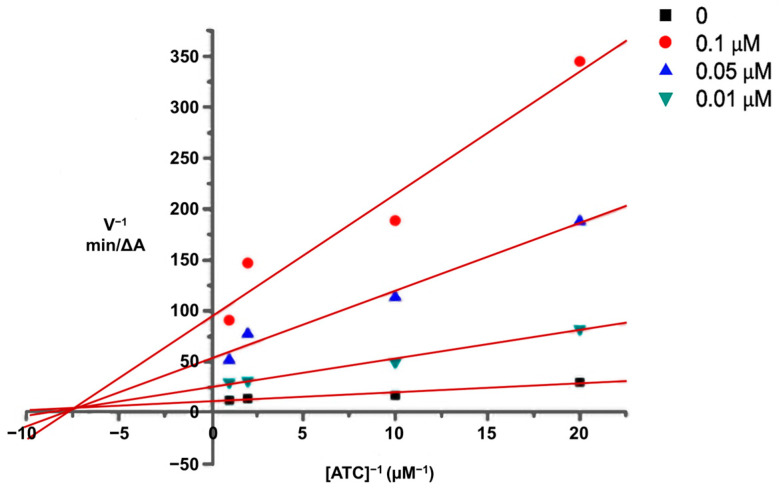
Lineweaver–Burk plot for the kinetic study of eeAChE’s inhibition by compound **16e**. The slope (Km/Vmax) reflects the enzyme’s affinity for the substrate in the presence of the inhibitor, while the y-intercept (1/Vmax) indicates the maximum velocity of the reaction.

Lineweaver–Burk double reciprocal plots (1/V versus 1/[S]) were constructed and are shown in Figure 2. The plots displayed a series of straight lines, with both the slope (Km/Vmax) and the y-intercept (1/Vmax) increasing as the inhibitor concentration increased. The slope (Km/Vmax) reflects the apparent enzyme–substrate affinity, whereas the y-intercept (1/Vmax) corresponds to the maximum reaction velocity. The progressive increase in slope indicates an apparent rise in Km values in the presence of **16e**, suggesting a reduction in substrate affinity. Meanwhile, the increase in y-intercept demonstrates a decrease in Vmax, indicating reduced catalytic efficiency. Together, these observations support a mixed-type inhibition mechanism, in which compound **16e** interacts with both the catalytic active site (CAS) and the peripheral anionic site (PAS) of AChE. These findings are consistent with the dual-site binding hypothesis proposed in our design strategy.

### 2.5. Self-Induced Aβ_1–42_ Aggregation

Self-induced Aβ_1–42_ aggregation of all the compounds we obtained was also evaluated. Our experimental approach relies on in vitro assays with ThT to monitor and quantify the aggregation of Aβ_1–42_ synthetic peptide into fibrils to test the anti-amyloidogenic activity of the target compounds [48]. ThT is a histochemical dye that is known to bind to the peptide β-sheet conformation, which is the predominant secondary structure of amyloid-beta fibrils. The presence of fibrils can be monitored by the fluorescence emission of ThT, with the absorption peak at 435 nm and the emission peak at 485 nm. The inhibition studies were carried out upon incubating Aβ_1–42_ with and without the compounds. Curcumin, a known active natural product for inhibition of Aβ_1–42_ self-aggregation, was used as a reference compound. From the results summarized in Table 1, it could be seen that most of the compounds showed moderate to good potencies (up to 94.6% at 20 μM) relative to that of curcumin (58.3% at 20 μM). Except **15b** and **15c**, all the other compounds showed inhibition of Aβ aggregation. Especially, compounds **15d**, **16c**–**16e**, **16g**–**16h**, **16k** all showed very good inhibition. As mentioned before, the key interaction between AChE and Aβ seems to be located at the PAS, since selective CAS inhibitors do not decrease Aβ aggregation; so the Aβ inhibition results also indicate that the hybrids must be simultaneously binding to the CAS and PAS of AChE. In addition, a structure–activity relationship (SAR) comparison highlighted that 8-aminoquinoline derivatives (e.g., **15f**, **16d**, **16g**, and **16k**) generally exhibited stronger inhibitory activities against AChE and Aβ aggregation; however, they lacked reliable metal-chelating ability. In contrast, 8-hydroxyquinoline derivatives displayed a more balanced multitarget profile, combining potent cholinesterase inhibition, effective suppression of Aβ aggregation, and robust metal-chelating properties.

### 2.6. Metal-Chelating Properties

**Figure 3:**
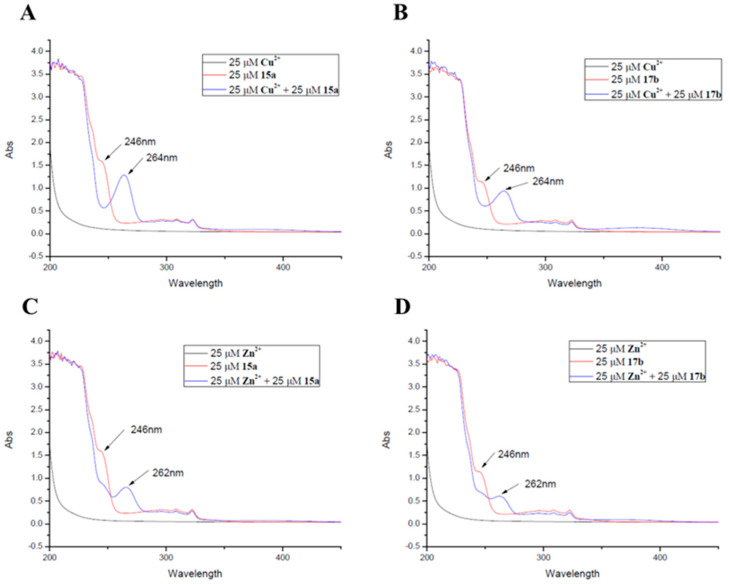
UV–vis absorption spectra of representative compounds and their metal ion complexes. (**A**,**C**) Complexation of compound **15a** with Cu^II^ and Zn^II^, showing red shifts of the maximum absorption peak from 246 nm to 264 nm and 262 nm, respectively. (**B**,**D**) Complexation of compound **17b** with Cu^II^ and Zn^II^, with the maximum absorption peak shifting from 246 nm to 265 nm and 262 nm, respectively.

Given the 8-hydroxy(amino)quinoline moiety with the properties to bind metal ions, the abilities of all the compounds in binding metal ions (Cu^II^, Fe^III^, Zn^II^) were studied by UV–vis spectrometry. At first three compounds were chose to test the binding abilities. Upon the addition of CuCl_2_ or ZnSO_4_ the maximum absorption suffered a bathochromic shift, pointing out the formation of complex compound–Cu^II^. As shown in Figure 3, the **15a**-Cu^II^ complex showed a maximum absorption in 264 nm that suffered a bathochromic shift from 246 nm, while the maximum absorption of **17b**-Cu^II^ complex changed from 246 nm to 265 nm; the **15a**-Zn^II^ complex showed a maximum absorption in 266 nm that suffered a bathochromic shift from 246 nm, while the maximum absorption of the **17b**-Zn^II^ complex changed from 246 nm to 262 nm; the significant changes in the maximum absorption indicated that the complex was formed from compounds and metal ions. Unfortunately, no reliable shift was found when Cu^II^ and Zn^II^ were added to the solution of compounds **16g**, suggesting that 8-aminoquinoline might not be a good metal-chelating moiety in these tacrine–qunoline hybrids. In addition, when Fe^III^ was added to all three types of solution **15a**, **16g**, and **17b**, no reliable shift was found, indicated that all three compounds could not form a complex with ferric ion.

**Figure 4:**
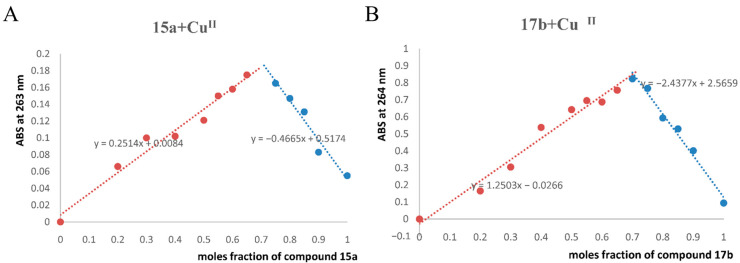
Determination of the stoichiometry of complexes **15a**-Cu^II^ (**A**) and **17b**-Cu^II^. (**B**) By Job’s method.

To determine the stoichiometry of the complex compound–Cu^II^, the technique of continuous variations (also called Job’s method) [49] was used by preparing solutions of compounds **15a**, **17b** and CuSO_4_ so that the sum of concentrations of both species was constant at 20 μm in all samples, but the proportions of both components varied between 0 and 100%. The UV spectra were recorded and the absorbance change at the maximum absorption wavelength of the compound-Cu^II^ complex, meaning the **15a**-Cu^II^ complex at 264 nm and the **17b**-Cu^II^ at 265 nm, respectively. The absorbance change was plotted versus the mole fraction of the compound, giving two straight lines whose equations were calculated. The equations gave a solution at a mole fraction around 0.7 for compounds **15a** and **17b**, revealing a 2:1 stoichiometry for complexes **15a**-Cu^II^ and **17b**-Cu^II^ (Figure 4). After this, the stoichiometry of all the other compounds was tested, and the results showed that all the tacrine–8-hydroxyquinoline hybrids could form complexes with Cu^II^ or Zn^II^ in a 2:1 stoichiometry (**15a**–**15e**, **16a**–**16c**, **16e**–**16f**, **16h**–**16j**, **17a**–**17b**), while tacrine–8-aminoquinoline hybrids with no binding properties to Cu^II^ or Zn^II^ (**15d**, **16d**, **16g**, **16k**).

After the determination of the stoichiometry of tacrine–8-hydroxyquinoline hybrids–Cu^II^ (Zn^II^), the affinity of compounds **15**–**17** as ligands (excepting **15f**, **16d**, **16g,** and **16k**) for Cu^II^ or Zn^II^ were estimated in a CH_3_OH/H_2_O solution (*v*/*v* = 9/1). Since all the ligands studied L from ML_2_ species and in accordance with the Beer–Lambert law, complexation reactions can be summarized as $2L+M\Leftrightarrow ML_{2}$ with K = $\frac{1-\alpha }{4C^{2}\alpha^{3}}$, and *α* can be calculated from the data of the absorption. All the binding affinities of the ligands with ions are listed in Table 2. As is shown, most of the compounds exhibited a good ability to bind the metal ions, with the affinity similar to clioquinol, and all showed no significant selectivity on Cu^II^ and Zn^II^.

### 2.7. Molecular Docking into Cholinesterases

To gain insights into the binding modes of the most potent compounds with AChE, molecular docking simulations were performed using the crystal structure of human AChE (PDB ID: 4EY7). Docking calculations were carried out using Schrödinger 2023-3 software, and the resulting complexes were visualized with PyMOL 3.1.6.1. Compounds **16e** and **16h** were selected as representative ligands based on their potent dual ChE inhibition and balanced biological profiles.

**Figure 5:**
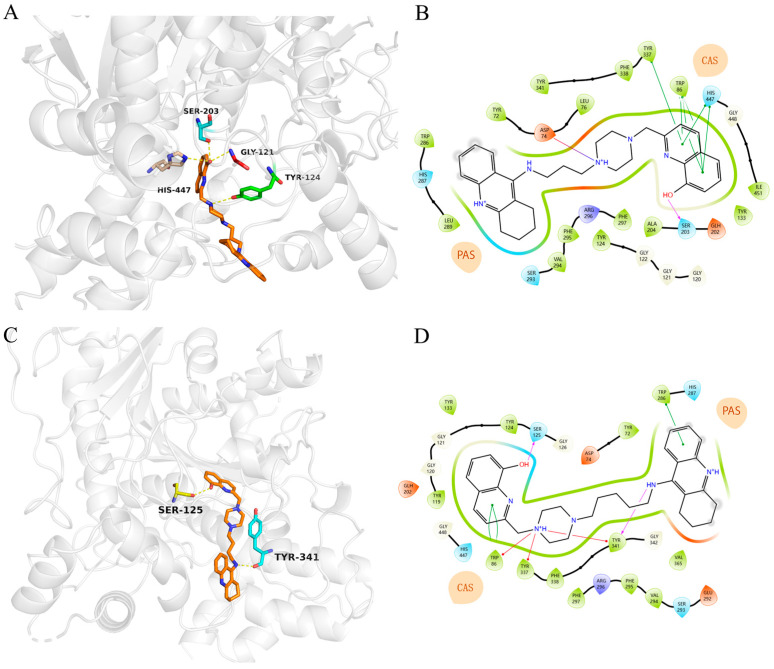
Molecular docking poses of compounds **16e** and **16h** with acetylcholinesterase (AChE, PDB ID: 4EY7): (**A**) 3D binding mode of compound **16e** at the catalytic active site (CAS) and peripheral anionic site (PAS) of AChE; (**B**) 2D schematic representation of key interactions between **16e** and AChE residues within the CAS and PAS; (**C**) 3D binding mode of compound **16h** within the AChE gorge, highlighting interactions at both CAS and PAS; (**D**) 2D interaction diagram of compound **16h** with AChE, depicting hydrogen bonds, π–π stacking, and other key contacts. Compounds **16e** and **16h** are depicted as orange sticks. Docking was performed using Schrödinger 2023-3 software, and the visualization was generated with PyMOL 3.1.6.1.

As shown in Figure 5, both compounds were successfully accommodated within the 20 Å deep AChE gorge, occupying regions corresponding to the catalytic active site (CAS) and peripheral anionic site (PAS), thus supporting their potential for dual-site inhibition.

Compound **16e** exhibited a rich interaction network. In the 3D docking pose, it formed key hydrogen bonds with Ser203, Tyr124, Gly121, and His447, residues critically located within or near the catalytic triad, indicating a strong engagement at the CAS. These data suggest that **16e** spans both PAS and CAS, stabilizing its binding through hydrogen bonding, π–π stacking, and van der Waals forces. In contrast, compound **16h** adopted a slightly shifted binding orientation, with prominent hydrogen bonds formed with Ser125 and Tyr341. However, unlike **16e**, **16h** did not interact directly with Ser203, possibly explaining subtle differences in their enzymatic inhibition profiles. Compound **15a** mainly interacted with residues at the peripheral site of BuChE (e.g., PRO-285, SER-287), whereas **17a** extended deeper into the catalytic gorge and formed hydrogen bonds with residues near the catalytic triad (GLU-197, SER-198). This dual-site engagement of **17a** is consistent with its stronger BuChE inhibitory activity compared with the **15** series.

Overall, both ligands demonstrated effective dual-site engagement within the AChE gorge. The interaction pattern of **16e**, with strong anchoring at the catalytic triad, may contribute to its slightly superior inhibitory potency. These docking results align well with the kinetic studies and SAR observations, providing a structural rationale for the multitarget inhibition behavior of the tacrine–quinoline hybrids.

## 3. Materials and Methods

### 3.1. Chemistry

Analytical grade reagents were purchased from Sigma-Aldrich (Shanghai, China), Energy (Shanghai, China), and Aladdin (Shanghai, China) without further purification. Reaction progress was monitored using analytical thin-layer chromatography (TLC) on precoated silica gel GF254 (Qingdao Haiyang Chemical Plant, Qingdao, China) plates, and the spots were detected under UV light (254 nm). ^1^H NMR spectra(400 MHz) and ^13^C NMR spectra (100 MHz) were measured on a Bruker AVANCE III at 25 °C and referenced to TMS. Elemental analyses were carried out by a Perkin Elmer Series II elemental analyzer. The results of the elemental analyses (C, H, N) were within ± 0.4% of the calculated values.

#### 3.1.1. General Procedures for the Preparation of Compounds **15**–**16**

To a 25 mL round flask charged with a mixture of intermediates **5**/**9** (0.5 mmol, 1.0 equiv) and **12**/**13** (0.5 mmol, 1.0 equiv) in DCE (dry, 5 mL), NaBH(OAc)_3_ (1.0 mmol, 2.0 equiv) was added under a N_2_ atmosphere. The mixture was heated to 50 °C and stirred until the reaction was completed (monitored by TLC); after cooling to room temperature, water (10 mL) was added, and the mixture was extracted with DCM (10 mL × 3), the combined organic phase was dried over anhydrous Na_2_SO_4_ and concentrated under reduced pressure, the residue was purified by column chromatography on silicon gel (300–400 mesh) to afford the intermediates with protecting groups of Bn or Boc. Then the protecting groups were removed to provide compounds **15** and **16**. To remove the protecting group of Bn, 37% HCl (10 equiv) was added to the corresponding intermediates, and the mixture was heated to reflux for 1 h. After cooling to room temperature, water (10 mL) was added, and K_2_CO_3_ was added to adjust the pH value > 7; then the mixture was extracted with DCM (10 mL × 3), the combined organic phase was dried over anhydrous Na_2_SO_4_ and concentrated under reduced pressure, and the residue was purified by column chromatography on silicon gel (300–400 mesh) to afford the corresponding compound of **15**/**16**. To remove the protecting group of Boc, DCM/CF_3_CO_2_H (*v*/*v* = 1:1, 2 mL) was added to the corresponding intermediates, and the mixture was stirred under room temperature for 1 h; water (10 mL) was added, and K_2_CO_3_ was added to adjust the pH value > 7, then the mixture was extracted with DCM (10 mL × 3), the combined organic phase was dried over anhydrous Na_2_SO_4_ and concentrated under reduced pressure, and the residue was purified by column chromatography on silicon gel (300–400 mesh) to afford the corresponding compounds of **15f**, **16d**, **16g**, and **16k**.

*2-(4-((8-Hydroxyquinolin-2-yl)methyl)piperazin-1-yl)-N-(1,2,3,4-tetrahydroacridin-9-yl)acetamide (***15a***).* Yield 66%, White solid; ^1^H NMR (400 MHz, CDCl_3_) δ 9.22 (s, 1H), 8.14 (d, *J* = 10.0 Hz, 1H), 8.00 (d, *J* = 8.0 Hz, 1H), 7.71 (d, *J* = 8.4 Hz, 1H), 7.66–7.61 (m, 2H), 7.48–7.41 (m, 2H), 7.32 (d, *J* = 8.4 Hz, 1H), 7.16 (d, *J* = 7.6 Hz, 1H), 3.88 (s, 2H), 3.33 (s, 2H), 3.15 (t, *J* = 6.4 Hz, 2H), 2.85–2.80 (m, 6H), 2.70 (m, 4H), 1.99–1.95 (m, 2H), 1.90–1.85 ppm (m, 2H); ^13^C NMR (100 MHz, CDCl_3_) δ 168.6, 159.9, 156.8, 152.0, 147.0, 138.1, 137.5, 136.6, 129.0, 128.8, 127.6, 127.4, 127.1, 126.0, 123.7, 121.9, 121.8, 117.7, 110.1, 64.6, 61.8, 54.0, 53.5, 34.0, 25.8, 22.7, 22.5 ppm; ESI-MS *m/z*: 504.2 [M + Na]^+^.
*2-(4-((5-Chloro-8-hydroxyquinolin-2-yl)methyl)piperazin-1-yl)-N-(1,2,3,4-tetrahydroacridin-9-yl)acetamide (***15b***).* Yield 63%, White solid; ^1^H NMR (400 MHz, CDCl_3_) δ = 9.21 (s, 1H), 8.49 (d, *J* = 8.8 Hz, 1H), 8.00 (d, *J* = 8.4 Hz, 1H), 7.76–7.70 (m, 2H), 7.63 (t, *J* = 7.2 Hz, 1H), 7.49–7.45 (m, 2H), 7.09 (d, *J* = 8.8 Hz, 1H), 3.89 (s, 2H), 3.31 (s, 2H), 3.15 (t, *J* = 6.4 Hz, 2H), 2.85–2.80 (m, 6H), 2.70 (m, 4H), 2.01–1.97 (m, 2H), 1.88–1.86 ppm (m, 2H); ^13^C NMR (100 MHz, CDCl_3_) δ = 168.6, 159.9, 157.6, 151.2, 146.9, 138.1, 137.9, 133.9, 128.9, 128.8, 127.1, 126.0, 125.5, 123.7, 122.5, 121.8, 120.4, 110.2, 64.3, 61.8, 53.9, 53.5, 34.0, 25.8, 22.7, 22.5 ppm; ESI-MS *m/z*: 538.3 [M + Na]^+^.
*2-(4-((5-Fluoro-8-hydroxyquinolin-2-yl)methyl)piperazin-1-yl)-N-(1,2,3,4-tetrahydroacridin-9-yl)acetamide (***15c***).* Yield 60%, White solid; ^1^H NMR (400 MHz, CDCl_3_) δ 9.21 (s, 1H), 8.38 (d, *J* = 8.8 Hz, 1H), 8.00 (d, *J* = 8.4 Hz, 2H), 7.74–7.70 (m, 2H), 7.63 (t, *J* = 7.6 Hz, 1H), 7.47 (t, *J* = 7.6 Hz, 1H), 7.13–7.03 (m, 2H), 3.88 (s, 2H), 3.33 (s, 2H), 3.15 (t, *J* = 6.4 Hz, 2H), 2.86–2.80 (m, 6H), 2.71 (m, 4H), 2.01–1.97 (m, 2H), 1.90–1.86 ppm (m, 2H); ^13^C NMR (100 MHz, CDCl_3_) δ 168.6, 159.9, 158.0, 150.7 (*J_C-F_* = 244.1 Hz), 148.2 (*J_C-F_* = 2.7 Hz), 147.0, 138.0, 137.1, 137.0, 130.3 (*J_C-F_* = 3.1 Hz), 129.0, 128.9, 127.1, 125.9, 123.7, 121.8, 121.8 (*J_C-F_* = 1.9 Hz), 118.0 (*J_C-F_* = 18.7 Hz), 110.4 (*J_C-F_* = 20.5 Hz), 108.8 (*J_C-F_* = 7.1 Hz), 64.5, 61.8, 54.0, 53.5, 34.0, 25.8, 22.7, 22.5 ppm; ESI-MS *m/z*: 522.2 [M + Na]^+^.
*2-(4-((6-Chloro-8-hydroxyquinolin-2-yl)methyl)piperazin-1-yl)-N-(1,2,3,4-tetrahydroacridin-9-yl)acetamide (***15d***).* Yield 59%, pale yellow solid; ^1^H NMR (400 MHz, CDCl_3_) δ 9.21 (s, 1H), 8.04 (d, *J* = 8.4 Hz, 1H), 8.00 (d, *J* = 8.4 Hz, 1H), 7.71–7.761 (m, 3H), 7.49–7.46 (m, 1H), 7.31 (d, *J* = 2.0 Hz, 1H), 7.14 (d, *J* = 2.4 Hz, 1H), 3.85 (s, 2H), 3.33 (s, 2H), 3.15 (d, *J* = 6.4 Hz, 2H), 2.85–2.81 (m, 6H), 2.77–2.69 (m, 4H), 1.99–1.98 (m, 2H), 1.89–1.86 ppm (m, 2H); ^13^C NMR (100 MHz, CDCl_3_) δ 168.6, 159.9, 157.1, 152.8, 147.0, 138.1, 136.2, 135.8, 133.1, 129.0, 128.9, 127.9, 127.1, 126.0, 123.7, 122.9, 121.8, 116.5, 111.5, 64.5, 61.8, 54.0, 53.5, 34.0, 25.8, 22.7, 22.5 ppm; ESI-MS *m/z*: 538.3 [M + Na]^+^.
*2-(4-((5,7-Dichloro-8-hydroxyquinolin-2-yl)methyl)piperazin-1-yl)-N-(1,2,3,4-tetrahydroacridin-9-yl)acetamide (***15e***).* Yield 65%, pale yellow solid; ^1^H NMR (400 MHz, CDCl_3_) δ 9.19 (s, 1H), 8.46 (d, *J* = 8.8 Hz, 1H), 8.00 (d, *J* = 8.4 Hz, 1H), 7.76 (d, *J* = 8.8 Hz, 1H), 7.70 (d, *J* = 8.4 Hz, 1H), 7.63 (t, *J* = 8.0 Hz, 1H), 7.57 (s, 1H), 7.46 (t, *J* = 7.6 Hz, 1H), 3.88 (s, 2H), 3.31 (s, 2H), 3.15 (t, *J* = 6.4 Hz, 2H), 2.83–2.80 (m, 6H), 2.68 (m, 4H), 2.01–1.95 (m, 2H), 1.90–1.86 ppm (m, 2H); ^13^C NMR (100 MHz, CDCl_3_,) δ 168.6, 159.9, 158.9, 147.5, 146.9, 138.1, 137.9, 134.1, 129.0, 128.8, 127.9, 127.1, 126.0, 124.2, 123.7, 122.4, 121.8, 120.7, 115.5, 64.2, 61.8, 53.9, 53.5, 34.0, 25.8, 22.7, 22.5 ppm; ESI-MS *m/z*: 572.2 [M + Na]^+^.
*2-(4-((8-Aminoquinolin-2-yl)methyl)piperazin-1-yl)-N-(1,2,3,4-tetrahydroacridin-9-yl)acetamide (***15f***).* Yield 61%, pale yellow solid; ^1^H NMR (400 MHz, CDCl_3_) δ 9.24 (s, 1H), 8.04 (d, *J* = 8.4 Hz, 1H), 8.00 (d, *J* = 8.4 Hz, 1H), 7.71 (d, *J* = 8.0 Hz, 1H), 7.63 (t, *J* = 7.6 Hz, 1H), 7.57 (d, *J* = 8.4 Hz, 1H), 7.46 (t, *J* = 7.6 Hz, 1H), 7.30 (t, *J* = 7.6 Hz, 1H), 7.13 (d, *J* = 8.0 Hz, 1H), 6.91 (d, *J* = 7.2 Hz, 1H), 4.99 (m, 2H), 3.88 (s, 2H), 3.33 (s, 2H), 3.15 (t, *J* = 6.4 Hz, 2H), 2.85–2.80 (m, 6H), 2.80–2.72 (m, 4H), 2.00–1.94 (m, 2H), 1.89–1.85 ppm (m, 2H); ^13^C NMR (100 MHz, CDCl_3_,) δ 168.7, 159.9, 156.0, 147.0, 143.7, 138.1, 137.5, 136.5, 128.9, 128.8, 127.9, 127.1, 125.9, 123.7, 121.9, 121.2, 115.9, 110.2, 65.0, 61.9, 54.1, 53.4, 34.0, 25.8, 22.7, 22.5 ppm; ESI-MS *m/z*: 503.3 [M + Na]^+^.
*2-((4-(2-((1,2,3,4-Tetrahydroacridin-9-yl)amino)ethyl)piperazin-1-yl)methyl)quinolin-8-ol (***16a***).* Yield 55%, pale yellow oil; ^1^H NMR (400 MHz, CDCl_3_) δ 8.54 (d, *J* = 8.4 Hz, 1H), 8.18 (d, *J* = 8.8 Hz, 1H), 8.14 (d, *J* = 8.8 Hz, 1H), 7.68 (t, *J* = 7.2 Hz, 1H), 7.62 (d, *J* = 8.8 Hz, 1H), 7.46–7.40 (m, 2H), 7.34–7.31 (m, 1H), 7.18–7.16 (m, 1H), 7.00 (m, 1H), 4.05–3.94 (m, 2H), 3.90 (s, 2H), 3.38–3.26 (m, 2H), 2.81 (t, *J* = 5.2 Hz, 2H), 2.76–2.67 (m, 8H), 2.57 (t, *J* = 5.2 Hz, 2H), 1.95–1.89 ppm (m, 4H); ^13^C NMR (100 MHz, CDCl_3_) δ 155.6, 153.8, 151.0, 150.9, 138.5, 136.4, 135.6, 131.1, 126.5, 126.4, 123.9, 122.9, 120.9, 120.8, 116.6, 115.0, 109.9, 109.1, 63.6, 55.0, 52.5, 51.3, 42.5, 27.6, 22.5, 21.0, 19.9 ppm; ESI-MS *m/z*: 468.3 [M + H]^+^.
*5-Fluoro-2-((4-(2-((1,2,3,4-tetrahydroacridin-9-yl)amino)ethyl)piperazin-1-yl)methyl)quinolin-8-ol (***16b***).* Yield 51%, pale yellow oil; ^1^H NMR (400 MHz, CDCl_3_) δ 8.36 (d, *J* = 8.4 Hz, 1H), 8.04 (d, *J* = 8.0 Hz, 1H), 7.99 (d, *J* = 8.4 Hz, 1H), 7.70 (d, *J* = 8.4 Hz, 1H), 7.58–7.54 (m, 1H), 7.36–7.32 (m, 1H), 7.13–7.04 (m, 2H), 5.39 (m, 1H), 3.88 (s, 2H), 3.61 (t, *J* = 4.8 Hz, 2H), 3.15–3.05 (m, 2H), 2.80–2.75 (m, 2H), 2.64–2.59 (m, 10H), 1.93–1.91 ppm (m, 4H); ^13^C NMR (100 MHz, CDCl_3_) δ 158.1, 157.7, 151.5, 150.7 (*J*_C-F_ = 244.3 Hz), 149.5, 148.3 (*J*_C-F_ = 3.2 Hz), 146.4, 137.1 (*J*_C-F_ = 2.6 Hz), 130.3 (*J*_C-F_ = 3.5 Hz), 128.8, 123.7, 122.9, 121.8 (*J*_C-F_ = 2.2 Hz), 119.8, 117.9 (*J*_C-F_ = 15.8 Hz), 115.4, 110.4 (*J*_C-F_ = 20.1 Hz), 108.8 (*J*_C-F_ = 7.7 Hz), 64.7, 57.4, 53.6, 52.6, 45.0, 33.3, 24.8, 23.0, 22.6 ppm; ESI-MS *m/z*: 486.3 [M + H]^+^.
*5,7-Dichloro-2-((4-(2-((1,2,3,4-tetrahydroacridin-9-yl)amino)ethyl)piperazin-1-yl)methyl)quinolin-8-ol (***16c***).* Yield 56%, pale yellow oil; ^1^H NMR (400 MHz, CDCl_3_) δ 8.45–8.41 (m, 1H), 8.04–7.99 (m, 2H), 7.74–7.70 (m, 1H), 7.58–7.55 (m, 2H), 7.36–7.32 (m, 1H), 5.47 (m, 1H), 3.87–3.85 (m, 2H), 3.70–3.55 (m, 2H), 3.17–3.03 (m, 2H), 2.80–2.73 (m, 2H), 2.64–2.63 (m, 10H), 1.98–1.82 ppm (m, 4H); ^13^C NMR (100 MHz, CDCl_3_) δ 158.9, 157.2, 151.8, 147.8, 145.9, 138.1, 133.9, 129.0, 127.8, 127.4, 124.1, 123.8, 123.0, 122.5, 120.4, 119.5, 115.5, 115.1, 64.3, 57.3, 53.6, 52.5, 44.9, 32.9, 24.7, 23.0, 22.5 ppm; ESI-MS *m/z*: 536.2 [M + H]^+^.
*N-(2-(4-((8-aminoquinolin-2-yl)methyl)piperazin-1-yl)ethyl)-1,2,3,4-tetrahydroacridin-9-amine (***16d***).* Yield 49%, colorless oil; ^1^H NMR (400 MHz, CDCl_3_) δ 8.48 (d, *J* = 8.4 Hz, 1H), 8.10 (d, *J* = 8.8 Hz, 1H), 7.97 (d, *J* = 8.4 Hz, 1H), 7.62–7.58 (m, 1H), 7.48 (d, *J* = 8.4 Hz, 1H), 7.36–7.32 (m, 1H), 7.23 (t, *J* = 8.0 Hz, 1H), 7.08–7.05 (m, 1H), 7.02 (m, 1H), 6.87–6.85 (m, 1H), 3.92 (t, *J* = 5.2 Hz, 2H), 3.83 (s, 2H), 3.24 (t, *J* = 5.2 Hz, 2H), 2.74 (t, *J* = 5.6 Hz, 2H), 2.70–2.52 (m, 8H), 2.48 (t, *J* = 6.0 Hz, 2H), 1.85–1.78 ppm (m, 4H); ^13^C NMR (100 MHz, CDCl_3_) δ 155.5, 155.0, 151.6, 143.7, 139.3, 137.5, 136.4, 132.2, 127.8, 127.2, 125.0, 123.9, 121.5, 121.3, 115.9, 115.8, 110.8, 110.2, 64.9, 56.0, 53.4, 52.3, 43.5, 28.4, 23.5, 22.0, 20.8 ppm; ESI-MS *m/z*: 467.3 [M + H]^+^.
*2-((4-(3-((1,2,3,4-Tetrahydroacridin-9-yl)amino)propyl)piperazin-1-yl)methyl)quinolin-8-ol (***16e***).* Yield 53%, colorless oil; ^1^H NMR (400 MHz, CDCl_3_) δ 8.39 (d, *J* = 8.4 Hz, 1H), 8.15 (d, *J* = 8.4 Hz, 1H), 8.06 (d, *J* = 8.4 Hz, 1H), 7.69 (m, 1H), 7.57–7.52 (m, 2H), 7.35 (t, *J* = 8.0 Hz, 1H), 7.30–7.23 (m, 2H), 7.10–7.08 (m, 1H), 4.00 (t, *J* = 5.2 Hz, 2H), 3.81 (s, 2H), 3.26–3.12 (m, 2H), 2.65–2.60 (m, 10H), 2.51–2.50 (m, 2H), 1.90 (t, *J* = 4.8 Hz, 2H), 1.77–1.76 ppm (m, 4H); ^13^C NMR (100 MHz, CDCl_3_) δ 156.4, 155.4, 152.0, 151.4, 139.6, 137.5, 136.6, 131.8, 127.6, 127.5, 124.6, 124.4, 121.9, 121.5, 117.7, 116.1, 110.9, 110.2, 64.7, 58.3, 53.9, 52.7, 50.0, 28.7, 25.6, 25.4, 21.8, 20.9 ppm; ESI-MS *m/z*: 482.3 [M + H]^+^.
*5-Fluoro-2-((4-(3-((1,2,3,4-tetrahydroacridin-9-yl)amino)propyl)piperazin-1-yl)methyl)quinolin-8-ol (***16f***).* Yield 59%, pale yellow oil; ^1^H NMR (400 MHz, CDCl_3_) δ 8.42 (d, *J* = 8.4 Hz, 1H), 8.30 (d, *J* = 8.4 Hz, 1H), 8.16 (d, *J* = 8.8 Hz, 1H), 7.74 (s, 1H), 7.62–7.56 (m, 2H), 7.31 (t, *J* = 8.0 Hz, 1H), 7.06–6.97 (m, 2H), 4.03 (t, *J* = 5.6 Hz, 2H), 3.83 (s, 2H), 3.22 (t, *J* = 5.6 Hz, 2H), 2.68–2.62 (m, 10H), 2.53 (t, *J* = 5.6 Hz, 2H), 1.92 (t, *J* = 4.8 Hz, 2H), 1.81–1.78 ppm (m, 4H); ^13^C NMR (100 MHz, CDCl_3_) δ 157.6, 155.5, 151.4, 150.7 (*J*_C-F_ = 244.7 Hz), 148.2 (*J*_C-F_ = 3.3 Hz), 139.5, 137.1 (*J*_C-F_ = 3.3 Hz), 132.0, 130.4 (*J*_C-F_ = 1.8 Hz), 124.7, 124.4, 121.8 (*J*_C-F_ = 3.0 Hz), 121.5, 117.9 (*J*_C-F_ = 18.8 Hz), 116.0, 110.8, 110.5 (*J*_C-F_ = 21.4 Hz), 108.9 (*J*_C-F_ = 7.4 Hz), 64.7, 58.4, 53.9, 52.7, 50.2, 29.7, 25.6, 25.3, 21.8, 20.8 ppm; ESI-MS *m/z*: 500.3 [M + H]^+^.
*N-(3-(4-((8-aminoquinolin-2-yl)methyl)piperazin-1-yl)propyl)-1,2,3,4-tetrahydroacridin-9-amine (***16g***).* Yield 61%, colorless oil; ^1^H NMR (400 MHz, CDCl_3_) δ 8.12 (d, *J* = 8.4 Hz, 1H), 8.04 (d, *J* = 8.4 Hz, 1H), 7.93 (d, *J* = 8.4 Hz, 1H), 7.48–7.43 (m, 2H), 7.24–7.17 (m, 2H), 7.02 (d, *J* = 8.0 Hz, 1H), 6.82 (d, *J* = 7.6 Hz, 1H), 6.72 (m, 1H), 4.97 (m, 2H), 3.77–3.72 (m, 4H), 3.13–3.07 (m, 2H), 2.68–2.35 (m, 12H), 1.82–1.79 (m, 2H), 1.77–1.73 ppm (m, 4H); ^13^C NMR (100 MHz, CDCl_3_) δ 155.7, 154.1, 153.7, 143.8, 142.7, 137.5, 136.3, 130.3, 127.8, 127.0, 124.2, 124.1, 124.0, 121.3, 117.7, 115.7, 112.8, 110.2, 65.1, 58.1, 53.8, 52.9, 49.6, 30.8, 26.1, 25.6, 22.2, 21.6 ppm; ESI-MS *m/z*: 481.3 [M + H]^+^.
*2-((4-(5-((1,2,3,4-Tetrahydroacridin-9-yl)amino)pentyl)piperazin-1-yl)methyl)quinolin-8-ol (***16h***).* Yield 55%, pale yellow oil; ^1^H NMR (400 MHz, CD_3_OD) δ 8.39 (d, *J* = 8.4 Hz, 1H), 8.22 (d, *J* = 8.8 Hz, 1H), 7.86–7.76 (m, 2H), 7.60–7.56 (m, 2H), 7.43–7.33 (m, 2H), 7.10–7.08 (m, 1H), 3.99–3.95 (m, 4H), 3.33–3.30 (m, 2H), 3.01–2.81 (m, 10H), 2.71–2.69 (m, 2H), 1.96–1.87 (m, 6H), 1.74–1.70 (m, 2H), 1.51–1.47 ppm (m, 2H); ^13^C NMR (100 MHz, CD_3_OD) δ 156.6, 155.3, 152.8, 150.5, 138.4, 137.9, 136.7, 132.7, 128.2, 127.2, 125.1, 125.0, 121.5, 118.8, 117.5, 115.8, 111.6, 110.8, 63.0, 57.0, 51.8, 50.9, 48.4, 29.7, 28.0, 24.3, 23.7, 23.6, 21.6, 20.5 ppm; ESI-MS *m/z*: 510.3 [M + H]^+^.
*5-Fluoro-2-((4-(5-((1,2,3,4-tetrahydroacridin-9-yl)amino)pentyl)piperazin-1-yl)methyl)quinolin-8-ol (***16i***).* Yield 57%, pale yellow oil; ^1^H NMR (400 MHz, CDCl_3_) δ 8.31 (d, *J* = 8.0 Hz, 1H), 8.26 (d, *J* = 8.8 Hz, 1H), 8.14 (d, *J* = 8.8 Hz, 1H), 7.58–7.52 (m, 2H), 7.34 (t, *J* = 8.0 Hz, 1H), 7.03–6.93 (m, 2H), 6.17 (m, 1H), 3.85 (t, *J* = 6.8 Hz, 2H), 3.81 (s, 2H), 3.23–3.10 (m, 2H), 2.72–2.57 (m, 12H), 1.82–1.77 (m, 6H), 1.66–1.61 (m, 2H), 1.46–1.42 ppm (m, 2H); ^13^C NMR (100 MHz, CDCl_3_) δ 156.3, 154.3, 150.8, 149.6 (*J*_C-F_ = 244.1 Hz), 147.5 (*J*_C-F_ = 3.1 Hz), 138.3, 136.2 (*J*_C-F_ = 3.3 Hz), 131.0, 129.3 (*J*_C-F_ = 2.7 Hz), 124.0, 123.2, 120.7 (*J*_C-F_ = 2.2 Hz), 120.3, 117.0 (*J*_C-F_ = 18.7 Hz), 115.1, 110.2, 109.5 (*J*_C-F_ = 21.0 Hz), 108.0 (*J*_C-F_ = 7.7 Hz), 63.1, 56.5, 51.6, 51.0, 47.0, 29.5, 27.8, 24.2, 23.2, 23.0, 21.0, 19.8 ppm; ESI-MS *m/z*: 528.3 [M + H]^+^.
*5-Chloro-2-((4-(5-((1,2,3,4-tetrahydroacridin-9-yl)amino)pentyl)piperazin-1-yl)methyl)quinolin-8-ol (***16j***).* Yield 58%, pale yellow oil; ^1^H NMR (400 MHz, CDCl_3_) δ 8.43 (d, *J* = 8.8 Hz, 1H), 8.38 (d, *J* = 8.8 Hz, 1H), 8.23 (d, *J* = 8.8 Hz, 1H), 7.68–7.60 (m, 2H), 7.45–7.38 (m, 2H), 7.05 (d, *J* = 8.4 Hz, 1H), 6.38 (m, 1H), 3.97–3.90 (m, 2H), 3.86 (s, 2H), 3.24–3.23 (m, 2H), 2.68–2.66 (m, 10H), 2.52 (t, *J* = 6.8 Hz, 2H), 1.89–1.86 (m, 6H), 1.64–1.62 (m, 2H), 1.50–1.47 ppm (m, 2H); ^13^C NMR (100 MHz, CDCl_3_) δ 157.4, 155.1, 151.9, 151.4, 139.7, 138.0, 133.8, 131.7, 127.0, 125.4, 124.9, 124.3, 122.5, 121.5, 120.2, 116.4, 111.5, 110.3, 64.1, 57.8, 52.8, 52.6, 48.1, 30.7, 29.1, 25.7, 24.4, 24.2, 22.1, 20.9 ppm; ESI-MS *m/z*: 544.3 [M + H]^+^.
*N-(5-(4-((8-aminoquinolin-2-yl)methyl)piperazin-1-yl)pentyl)-1,2,3,4-tetrahydroacridin-9-amine (***16k***).* Yield 62%, colorless oil; ^1^H NMR (400 MHz, CDCl_3_) δ 8.30–8.25 (m, 1H), 8.11 (d, *J* = 8.8 Hz, 1H), 7.93–7.91 (m, 1H), 7.55–7.51 (m, 1H), 7.45–7.42 (m, 1H), 7.33–7.29 (m, 1H), 7.19–7.17 (m, 1H), 7.03–7.01 (m, 1H), 6.84–6.82 (m, 1H), 6.01 (m, 1H), 4.96 (m, 2H), 3.77–3.74 (m, 4H), 3.14–3.13 (m, 2H), 2.58–2.34 (m, 12H), 1.78–1.76 (m, 6H), 1.54–1.49 (m, 2H), 1.41–1.38 ppm (m, 2H); ^13^C NMR (100 MHz, CDCl_3_) δ 155.9, 154.6, 152.6, 143.8, 140.5, 137.5, 136.3, 131.4, 127.8, 127.0, 124.8, 124.1, 122.3, 121.3, 116.7, 115.7, 111.9, 110.1, 64.9, 58.0, 53.1, 52.8, 48.3, 30.9, 29.5, 26.0, 24.5, 24.2, 22.2, 21.1 ppm; ESI-MS *m*/*z*: 509.3 [M + H]^+^.

#### 3.1.2. General Procedures for the Preparation of Compounds **17**

Intermediates **9** (1 mmol, 1.0 equiv) and **14** (1 mmol, 1.0 equiv) were dissolved in DCM (10 mL), and K_2_CO_3_ (1.0 mmol, 1.0 equiv) was added. And the mixture was stirred under room temperature until the reaction was completed (monitored by TLC); water (10 mL) was added, and the mixture was extracted with DCM (15 mL × 3); the combined organic phase was dried over anhydrous Na_2_SO_4_ and concentrated under reduced pressure to give the residue; the pure products could be obtained by recrystallization from hexane/ethyl estate.

*2-(4-((8-Hydroxyquinolin-5-yl)methyl)piperazin-1-yl)-N-(1,2,3,4-tetrahydroacridin-9-yl)acetamide (***17a***).* Yield 92%, pale yellow solid; ^1^H NMR (400 MHz, CDCl_3_) δ 9.26 (s, 1H), 8.80 (d, *J* = 3.2 Hz, 1H), 8.67 (d, *J* = 8.0 Hz, 1H), 8.01 (d, *J* = 8.4 Hz, 1H), 7.72 (d, *J* = 8.0 Hz, 1H), 7.64 (t, *J* = 7.2 Hz, 1H), 7.49–7.46 (m, 2H), 7.33 (d, *J* = 7.6 Hz, 1H), 7.08 (d, *J* = 8.0 Hz, 1H), 3.85 (s, 2H), 3.30 (s, 2H), 3.15 (t, *J* = 6.4 Hz, 2H), 2.83–2.77 (m, 6H), 2.62 (m, 4H), 2.00–1.97 (m, 2H), 1.89–1.86 ppm (m, 2H); ^13^C NMR (100 MHz, CDCl_3_) δ 168.7, 160.0, 152.0, 147.7, 146.9, 138.8, 138.1, 134.0, 129.0, 128.95, 128.8, 127.9, 127.1, 125.9, 124.2, 123.7, 121.9, 121.5, 108.6, 61.8, 60.6, 54.1, 53.1, 34.0, 25.8, 22.7, 22.5 ppm; ESI-MS *m*/*z*: 504.3 [M + Na]^+^.
*2-(4-((8-Hydroxy-2-methylquinolin-5-yl)methyl)piperazin-1-yl)-N-(1,2,3,4-tetrahydroacridin-9-yl)acetamide (***17b***).* Yield 93%, pale yellow solid; ^1^H NMR (400 MHz, CDCl_3_) δ 9.26 (s, 1H), 8.53 (d, *J* = 8.4 Hz, 1H), 8.00 (d, *J* = 8.0 Hz, 1H), 7.71 (d, *J* = 8.4 Hz, 1H), 7.65–7.61 (m, 1H), 7.49–7.45 (m, 1H), 7.34 (d, *J* = 8.8 Hz, 1H), 7.25 (d, *J* = 7.6 Hz, 1H), 7.03 (d, *J* = 7.6 Hz, 1H), 3.82 (s, 2H), 3.29 (s, 2H), 3.15 (t, *J* = 6.4 Hz, 2H), 2.82 (t, *J* = 6.4 Hz, 2H), 2.76 (m, 4H), 2.73 (s, 3H), 2.60 (m, 4H), 2.00–1.97 (m, 2H), 1.89–1.86 ppm (m, 2H); ^13^C NMR (100 MHz, CDCl_3_) δ 168.7, 160.0, 156.6, 151.5, 147.0, 138.13, 138.11, 134.1, 129.0, 128.8, 128.0, 127.1, 125.9, 123.9, 123.7, 122.4, 121.9, 108.5, 61.8, 60.6, 54.1, 53.1, 34.0, 25.8, 24.9, 22.7, 22.6 ppm; ESI-MS *m*/*z*: 518.3 [M + Na]^+^.

### 3.2. Biological Evaluation

#### 3.2.1. Inhibition Experiments of ChEs

Cholinesterase (ChE) inhibitory activity was evaluated using a modified Ellman’s spectrophotometric method. Acetylcholinesterase (AChE, from electric eel), butyrylcholinesterase (BuChE, from equine serum), 5,5′-dithiobis-(2-nitrobenzoic acid) (Ellman’s reagent, DTNB), *S*-butyrylthiocholine iodide (BTCI), acetylthiocholine iodide (ATCI), and tarcine hydrochloride were purchased from Sigma-Aldrich. Tarcine hydrochloride was used as a reference standard. All the assays were carried out in 0.1 M KH_2_PO_4_/K_2_HPO_4_ buffer (pH = 7.4). Stock solutions of tested compounds (10 mM) were prepared in DMSO (below 0.5%, *v*/*v*) and diluted in phosphate buffer. The assay was performed in 96-well plate by adding 25 μL of 1 mM acetylthiocholine iodide or butyrylthiocholine iodide used as substrate in the assay, 125 μL of 1 mM 5,5′-dithiobis-(2-nitrobenzoic acid) (DTNB), 25 μL of 0.1 M phosphate buffer (pH = 7.4), 25 μL of testing substance in various concentrations, and 50 μL of 0.2 Units/mL AChE from an electric eel or BuChE from equine serum, respectively. At least five concentrations of the test compounds were assayed. The absorbance changes at 405 nm were detected every 5 min over a period of 35 min with a microplate reader. The enzyme activity and the percentage inhibition were determined. The compound concentration producing 50% of ChE inhibition (IC_50_) was calculated graphically from a concentration–inhibition curve for each compound using Graphpad Prism 5.0.

#### 3.2.2. Kinetic Analysis of AChE Inhibition

To obtain a kinetic study of the mechanism of AChE inhibition by compound **16e**, Lineweaver–Burk double reciprocal plots of 1/velocity versus 1/[substrate] were constructed at a relatively low concentration of substrate (0.05–1 μM) by using Ellman’s method (Lineweaver–Burk equation 1/v = (Km/Vmax)(1/[S]) + 1/Vmax). Three concentrations of compound **16e** were selected for this study: 10, 50, and 100 nM. The assay medium contained 25 μL of PBS (pH 7.4), 50 μL of enzyme, 25 μL of compound solution, and 125 μL of 1.0 mM DTNB; the reaction was started by addition of 25 μL of different concentrations of ATC. Kinetic characterization of the hydrolysis of ATC catalyzed by AChE was measured at 415 nm at 3 min intervals by a microplate reader.

#### 3.2.3. Inhibition of Self-Induced Aβ_1–42_ Aggregation

Aβ_1–42_ was dissolved in 100% 1,1,1,3,3,3-hexafluoro-2-propanal (HFIP) to a concentration of 1 mg/mL, sonicated in a water bath overnight, aliquoted into microcentrifuge tubes, dried under vacuum, and stored at −20 °C. Immediately prior to use, the HFIP-treated Aβ_1–42_ was dissolved in dimethylsulfoxide (DMSO) as the stored solution. A screening assay for all compounds that inhibited Aβ_1–42_ aggregation was performed by measuring the ThT fluorescence emission. Amounts of 5 μL of compound (200 μM) and 5 μL of 200 μM Aβ_1–42_ were added into 40 μL of phosphate-buffered saline (PBS at pH 7.4). After incubation at room temperature on a thermostatic shaker for 72 h, 100 μL of 5 μM ThT solution (in PBS at pH 7.4) was added to the reaction solution. Fluorescence intensity was measured at 485 nm with an excitation wavelength of 435 nm on a microplate reader. The Aβ_1–42_ aggregation inhibition percentage was calculated using the following formula: inhibition percentage = (F_1_ − F_2_)/(F_1_ − F_0_) × 100%. (F_0_: fluorescence intensity of ThT solution, F_1_: fluorescence intensity of Aβ_1–42_ and ThT solution, F_2_: fluorescence intensity of solution added tacrine-quinoline hybrids, Aβ_1–42_ and ThT).

#### 3.2.4. Evaluation of Metal-Chelating Properties

Metal-chelating properties were evaluated by UV–vis spectrometry. To screen the ability of chelating metal ions, the same concentrations of compound and metal ion were mixed in CH_3_OH/H_2_O (*v*/*v* = 9/1), and the UV–vis spectrum was tested; compared to the compound, the maximum absorption that suffered a bathochromic shift would point out the formation of a complex compound–metal ion. The stoichiometry of the complex compound–metal ion was determined by Job’s method. The affinity of compounds **15**–**17** as ligands (excepted **15f**, **16d**, **16g**, **16k**) for Cu^II^ or Zn^II^ were estimated in the CH_3_OH/H_2_O solution (*v*/*v* = 9/1). Since all the ligands studied L from ML_2_ species and in accordance with the Beer–Lambert law, complexation reactions can be summarized as $2L+M\Leftrightarrow ML_{2}$ with K = $\frac{1-\alpha }{4C^{2}\alpha^{3}}$, and α = A/A_max_; A was the data of absorption tested when the compound–metal ion = 2/1 under the maximum absorption wavelength of the complex, A_max_ was the absorption when the compound completely formed a complex with the metal ion; the data can be obtained as an approximation when the metal ion is in excess, and the absorption changes little as the concentration of metal ion still increases.

#### 3.2.5. Molecular Docking Studies

Molecular docking was performed using Schrödinger Suite 2023-3 (Glide SP/XP modes) to evaluate the binding interactions of tacrine–quinoline hybrids with acetylcholinesterase (AChE, PDB ID: 4EY7). Protein preparation was carried out with the Protein Preparation Wizard in Maestro, in which bond orders were assigned, hydrogen atoms were added, water molecules beyond 5 Å from heteroatoms were removed, and the structure was minimized using the OPLS_2005 force field. Ligands were prepared with LigPrep at pH 7.0 ± 0.2 (Epik ionization states). The receptor grid was generated to encompass the active-site gorge, including both the catalytic active site (CAS) and the peripheral anionic site (PAS), with a grid box size of 20 × 20 × 20 Å and default van der Waals scaling parameters (scaling factor 1.0, partial charge cutoff 0.25). Docking calculations were performed in both SP and XP precision, and the best-ranked poses were selected according to the Glide docking score and consistency with known pharmacophore interactions. Visualization and analysis of docking poses were conducted using PyMOL 3.1.6.1 and Maestro.

## 4. Conclusions

In conclusion, a series of tarine–quinoline hybrids have been designed, synthesized and evaluated as novel multifunctional anti-AD agents. ChE inhibitory activities, Aβ-reducing activity, and metal-chelating properties were investigated; the results showed that most of the compounds had good activities against both AChE and BuChE, good inhibition of Aβ aggregation, and non-selective chelating properties on Cu^II^ and Zn^II^. Among the three series of compounds **15**–**17**, compounds **16** (except **16c**) showed the best activities against both AChE and BuChE in the submicromolar range in vitro. A kinetic study using model compound **16e** implied that compounds **16** could bind simultaneously to the PAS and CAS of AChE. In addition, UV–vis spectrometry analysis revealed that the 8-hydroxyquinoline moiety in these hybrids exhibited non-selective chelating properties to Cu^II^ and Zn^II^, while compounds with an 8-aminoquinoline moiety showing a poor binding ability to metal ions under the conditions used. The stoichiometry of the ligand–metal ion complexes and the affinity of the ligands were estimated. Overall, our preliminary results showed that the targeted compound tacrine–8-hydroxyquinoline hybrids exhibited a multifunctional effect against AD, including inhibitory activity on ChEs and Aβ aggregation and properties of chelating metal ions preferable to tacrine-8-aminoquinoline hybrids, which qualified them as potential anti-AD drug candidates. Notably, compounds **16e** and **16h** demonstrated the most favorable balanced activity against cholinesterases (ChEs), self-induced Aβ aggregation, and metal ion chelation. These findings indicate that these compounds could serve as excellent multifunctional anti-AD agents, and further investigations in cell and animal models are currently underway in our laboratory.

## Figures and Tables

**Table 1:** Inhibitory activity against AChE and BuChE, selectivity index, and self–induced Aβ_1–42_ aggregation.

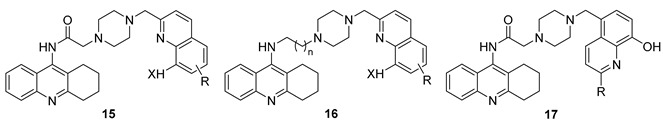					
Compd.	X, R, *n*	IC_50_ (μM) ^a^		Selectivity Index ^b^	Aβ Aggregation Inhibition (%) ^c^
		AChE Inhibition	BuChE Inhibition		
**15a**	X = O, R = H	2.02 ± 0.12	7.74 ± 0.89	0.26	46.8 ± 4.1
**15b**	X = O, R = 5-chloro	2.18 ± 0.17	11.45 ± 1.56	0.19	n.d
**15c**	X = O, R = 6-chloro	1.51 ± 0.31	11.98 ± 1.07	0.13	n.d
**15d**	X = O, R = 5-fluoro	1.18 ± 0.10	8.41 ± 1.41	0.14	88.7 ± 5.2
**15e**	X = O, R = 5,7-dichloro	5.73 ± 0.17	5.53 ± 0.27	1.04	55.9 ± 2.5
**15f**	X = NH, R = H	1.24 ± 0.21	7.85 ± 0.71	0.16	22.0 ± 1.8
**16a**	X = O, R = H, *n* = 1	0.44 ± 0.006	0.27 ± 0.004	1.63	77.0 ± 4.3
**16b**	X = O, R = 5-fluoro, *n* = 1	0.19 ± 0.005	0.20 ± 0.003	0.95	22.9 ± 2.4
**16c**	X = O, R = 5,7-dichloro, *n* = 1	2.68 ± 0.30	3.41 ± 0.006	0.79	92.5 ± 3.3
**16d**	X = NH, R = H, *n* = 1	0.18 ± 0.004	0.21 ± 0.003	0.86	84.7 ± 3.6
**16e**	X = O, R = H, *n* = 2	0.10 ± 0.0032	0.043 ± 0.005	2.33	80.5 ± 4.4
**16f**	X = O, R = 5-fluoro, *n* = 2	0.087 ± 0.002	0.028 ± 0.002	3.11	54.4 ± 2.9
**16g**	X = NH, R = H, *n* = 2	0.052 ± 0.001	0.018 ± 0.003	2.89	94.6 ± 4.2
**16h**	X = O, R = H, *n* = 4	0.21 ± 0.003	0.10 ± 0.01	2.10	93.2 ± 3.9
**16i**	X = O, R = 5-fluoro, *n* = 4	0.40 ± 0.006	0.12 ± 0.02	3.33	55.6 ± 5.3
**16j**	X = O, R = 5-chloro, *n* = 4	0.13 ± 0.02	0.045 ± 0.011	2.89	74.8 ± 4.7
**16k**	X = NH, R = H, *n* = 4	0.21 ± 0.003	0.054 ± 0.004	3.89	86.7 ± 3.8
**17a**	R = H	4.46 ± 0.33	4.13 ± 0.10	1.08	24.6 ± 3.7
**17b**	R = Me	2.53 ± 0.20	4.39 ± 0.27	0.58	23.3 ± 3.2
tacrine		0.17 ± 0.04	0.029 ± 0.005	5.86	–
curcumin		–	–		58.3 ± 2.5

^a^ Date are expressed as means ± SEM) of at least three experiments. ^b^ Selectivity index = IC_50_ (AChE)/IC_50_ (BuChE). ^c^ Inhibition of self-induced Aβ_1–42_ aggregation; the thioflavin-T fluorescence method was used, the mean ± SD of at least three independent experiments, and the measurements were carried out in the presence of 20 μM compounds.

**Table 2:** The affinity of compounds **15**–**17** as ligands for Cu^II^ or Zn^II^.

Comp.	LogK	
	Cu	Zn
**15a**	10.14	9.91
**15b**	10.09	11.34
**15c**	10.05	9.64
**15d**	10.08	9.81
**15e**	9.59	9.65
**15f**	-	-
**16a**	9.63	9.48
**16b**	9.99	9.63
**16c**	9.75	9.28
**16d**	-	-
**16e**	9.46	9.63
**16f**	9.62	9.45
**16g**	-	-
**16h**	9.87	9.63
**16i**	9.49	9.49
**16j**	9.96	9.65
**16k**	-	-
**17a**	9.50	9.67
**17b**	9.42	9.71
clioquinol	9.86 (10.08) ^a^	8.38 (8.84) ^a^

^a^ Data from the literature [50].

## Data Availability

Data are contained within the article and Supplementary Materials.

## Supplementary Materials

The following supporting information can be downloaded at: https://www.mdpi.com/article/10.3390/molecules30173489/s1, Supplementary data of ^1^H NMR and ^13^C NMR spectra of all the products to this article; Figure S1: Molecular docking poses of compounds 15a (A, B) and 17a (C, D) with BuChE (PDB ID:1POI).
