# Supplementary material for: Multifunctional Tacrine–Quinoline Hybrids as Cholinesterase Inhibitors, Aβ Aggregation Blockers, and Metal Chelators for Alzheimer’s Therapy
Source: Molecules. 2025 Aug 25;30(17):3489. doi: 10.3390/molecules30173489 (PMC12430544; doi:10.3390/molecules30173489)
Supplement: Supplementary file 1 [file molecules-30-03489-s001.zip › molecules-3825254-supplementary.pdf]

Multifunctional Tacrine–Quinoline Hybrids as Cholinesterase Inhibitors, Aβ Aggregation Blockers, and Metal Chelators for Alzheimer's Therapy

Xiaohua Wang [1], Minglan Ma [1], Yalan Feng [1], Jian Liu [2], Gang Wang [1 2] *

[1] Synergy Innovation Center of Biological Peptide Antidiabetics of Hubei Province, College of Life Science, Wuchang University of Technology, Wuhan, 430223，P.R. China. Email: msnhm123@gmail.com

[2] School of Chemistry and Environmental Engineering, Wuhan Institute of Technology, Wuhan, 430025, P.R. China. Email: wg@wit.edu.cn

[*] Corresponding author.

Table of contents

Representative Spectra of NMR 2

Molecular Docking 21

Representative Spectra of NMR

*2-(4-((8-Hydroxyquinolin-2-yl)methyl)piperazin-1-yl)-N-(1,2,3,4-tetrahydroacridin-9-yl)acetamide (**15a**)*

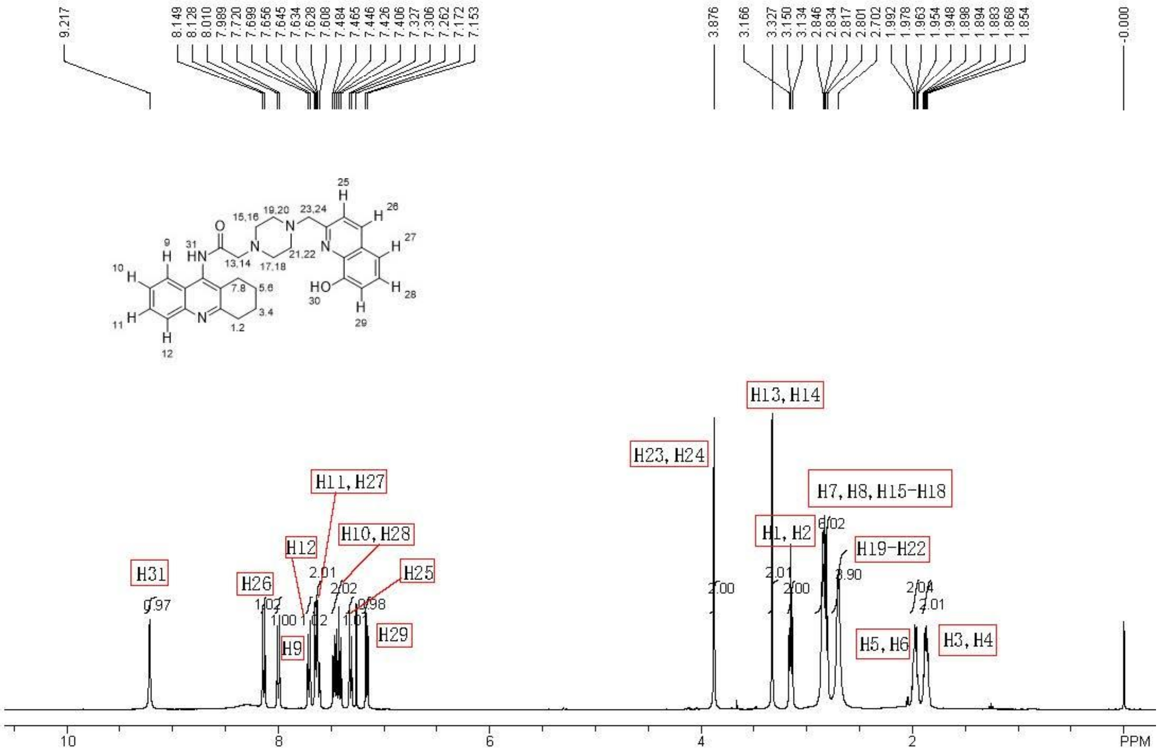

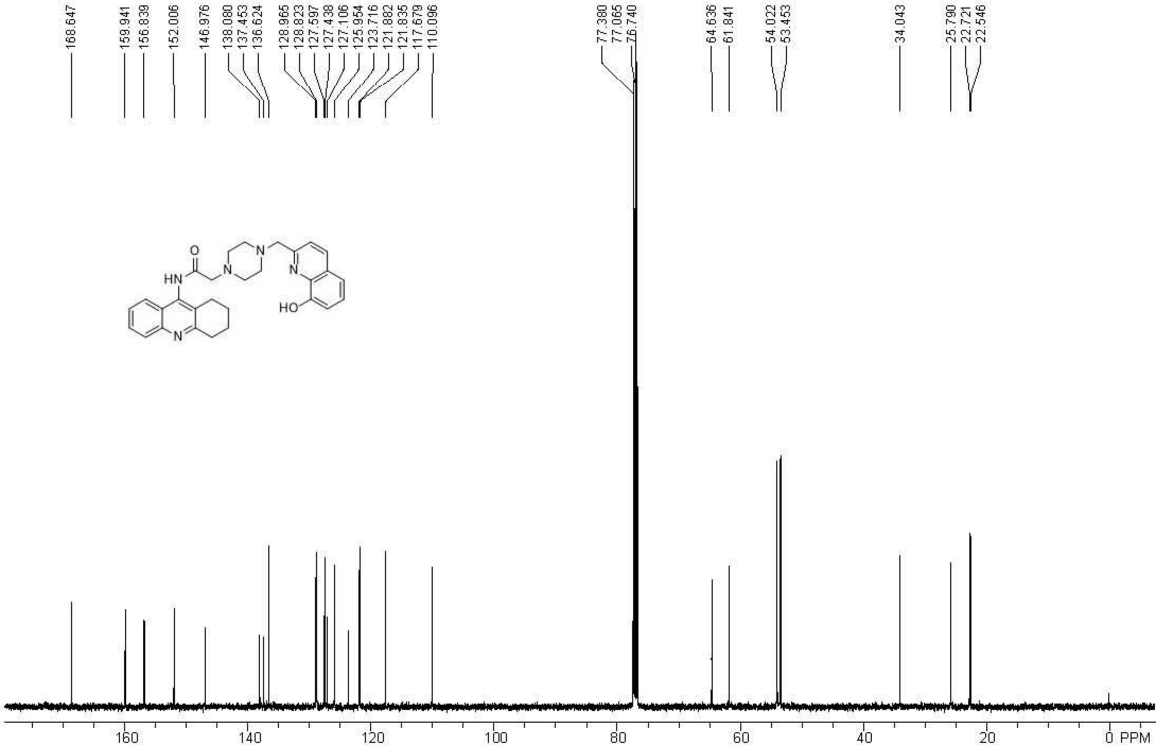

*2-(4-((5-Chloro-8-hydroxyquinolin-2-yl)methyl)piperazin-1-yl)-N-(1,2,3,4-tetrahydroacridin-9-yl)acetamide (**15b**)*

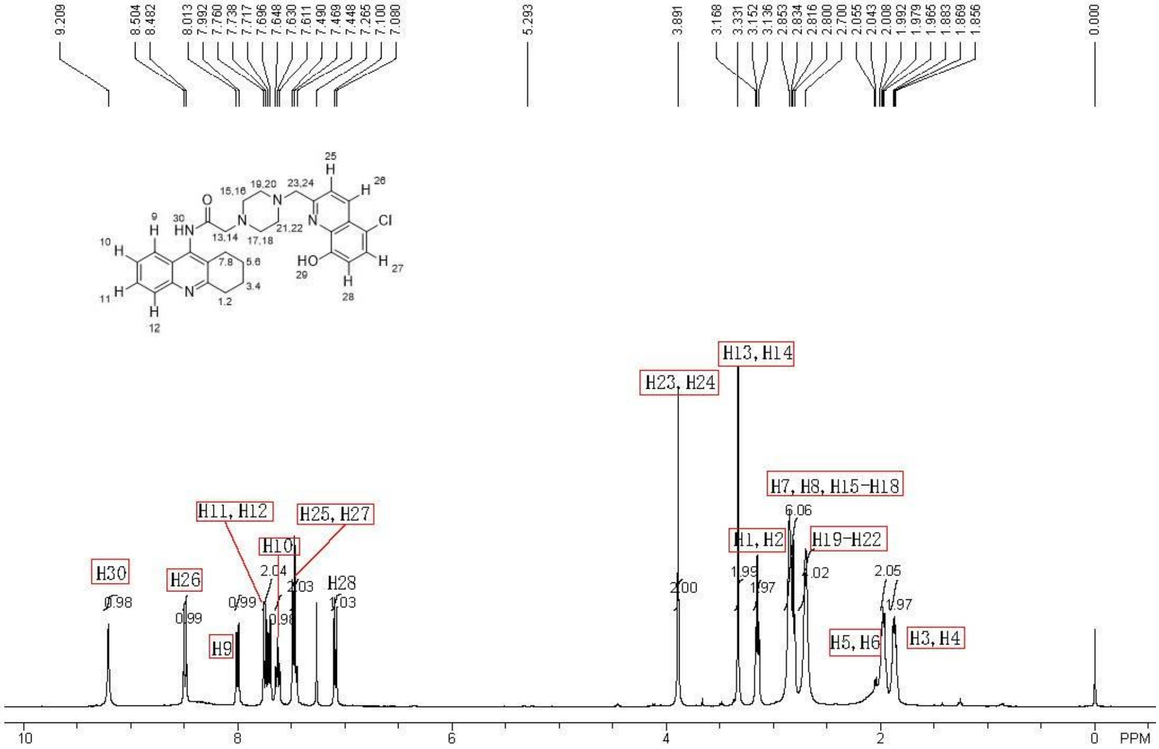

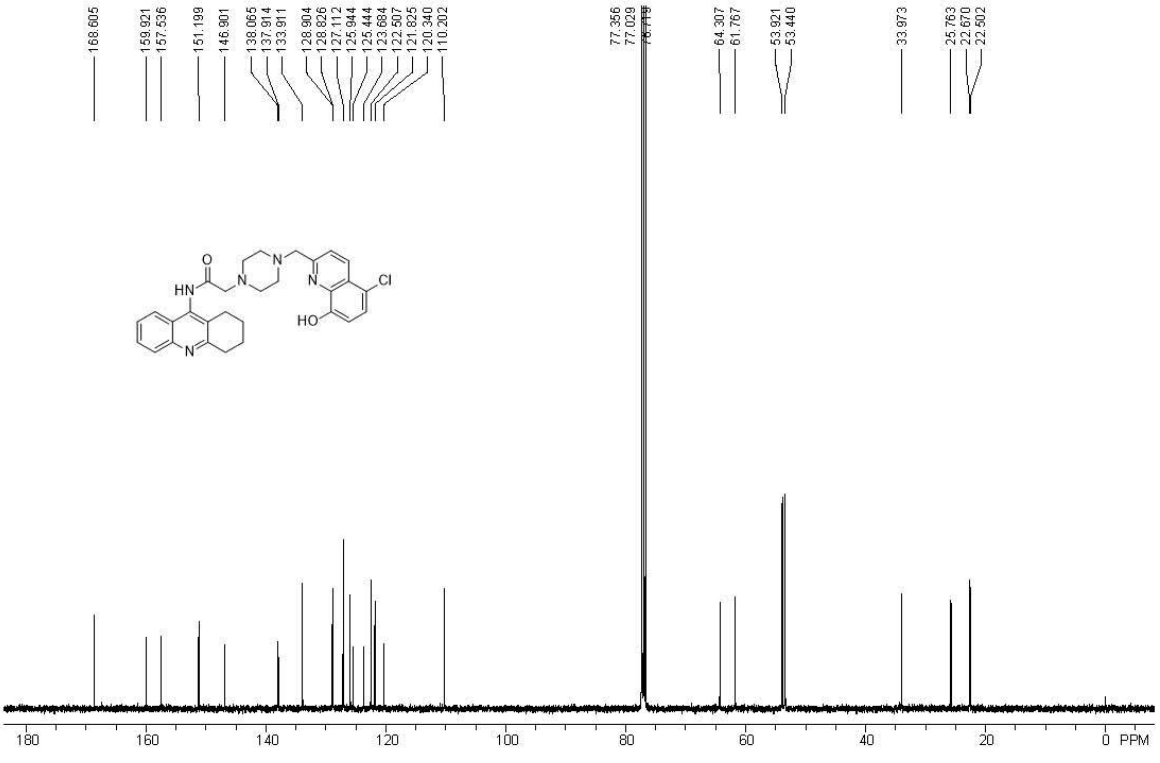

*2-(4-((5-Fluoro-8-hydroxyquinolin-2-yl)methyl)piperazin-1-yl)-N-(1,2,3,4-tetrahydroacridin-9-yl)acetamide (**15c**)*

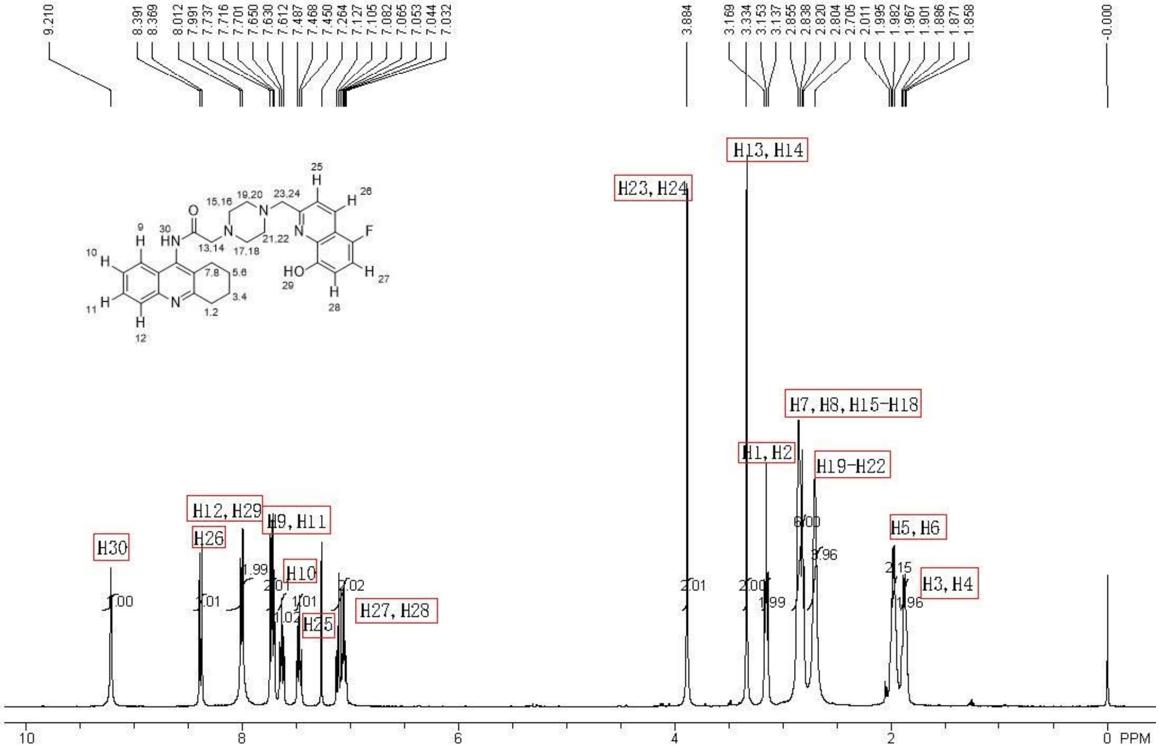

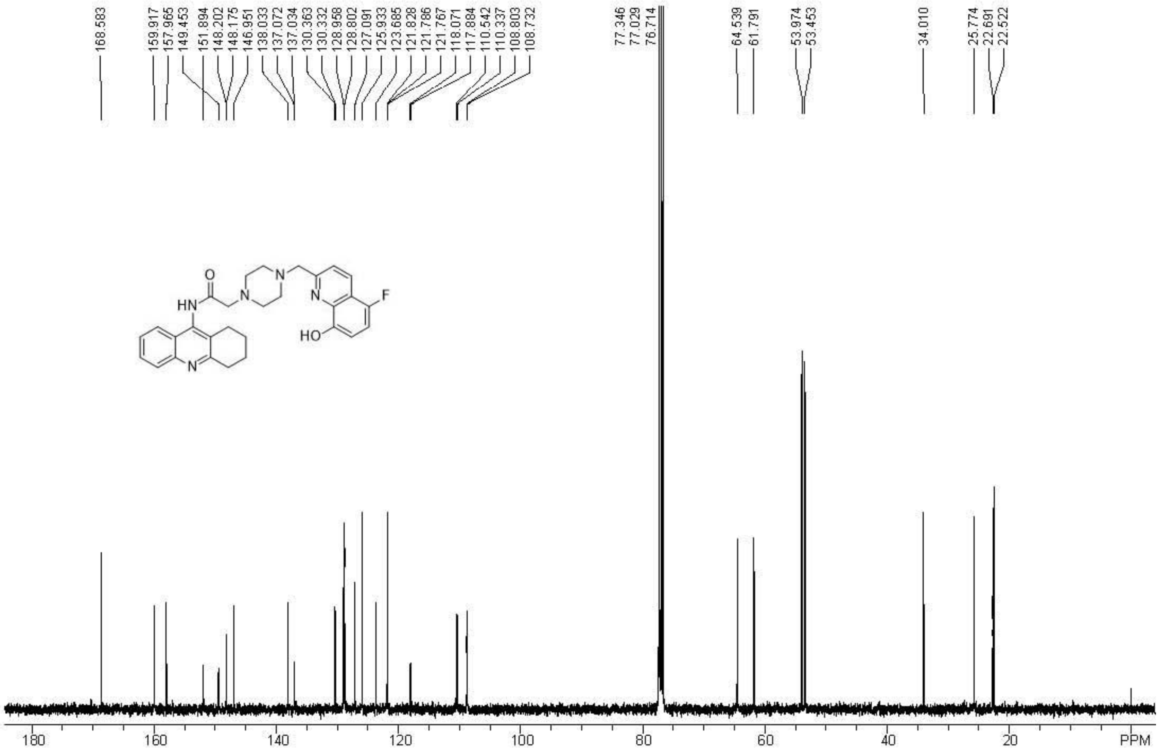

*2-(4-((6-Chloro-8-hydroxyquinolin-2-yl)methyl)piperazin-1-yl)-N-(1,2,3,4-tetrahydroacridin-9-yl)acetamide (**15d**)*

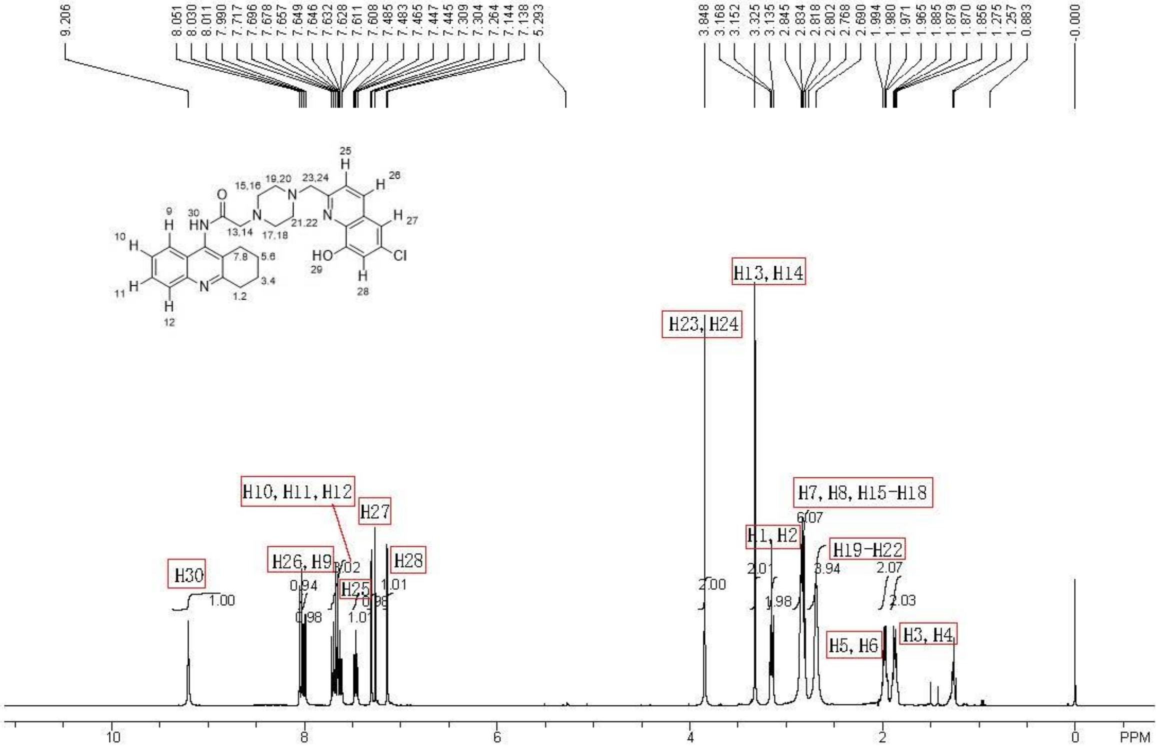

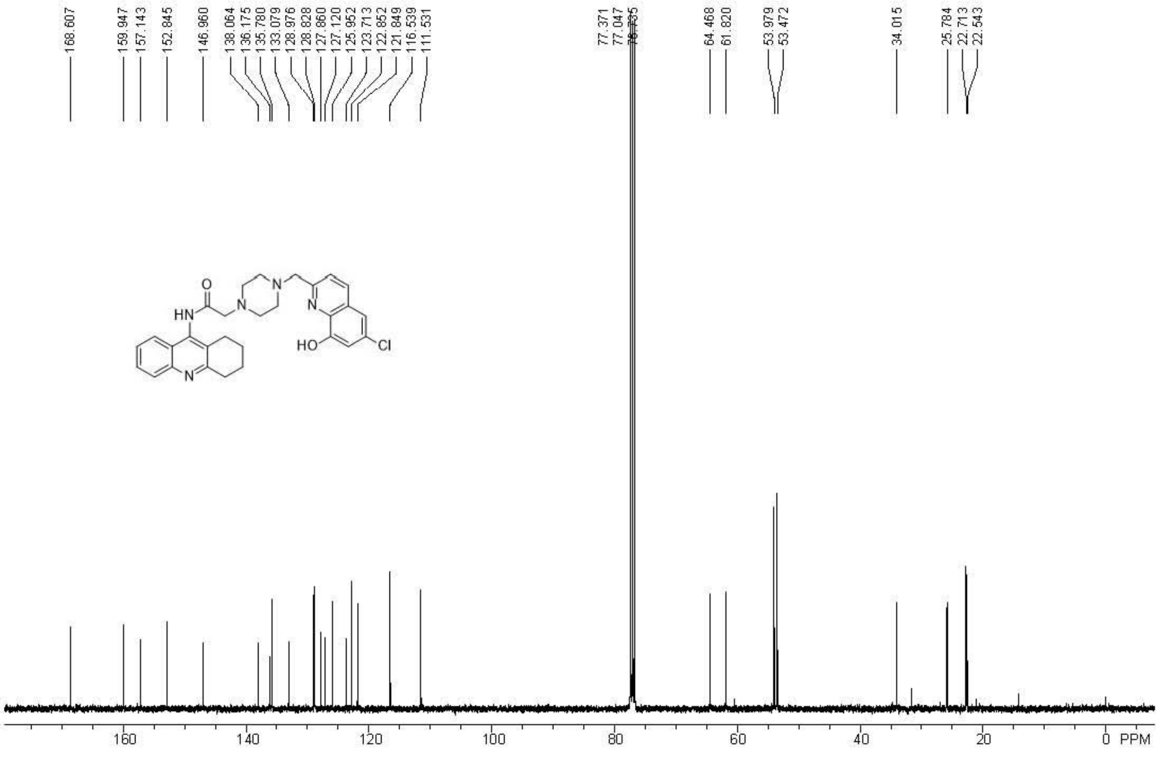

*2-(4-((5,7-Dichloro-8-hydroxyquinolin-2-yl)methyl)piperazin-1-yl)-N-(1,2,3,4-tetrahydroacridin-9-yl)ac etamide (**15e**)*

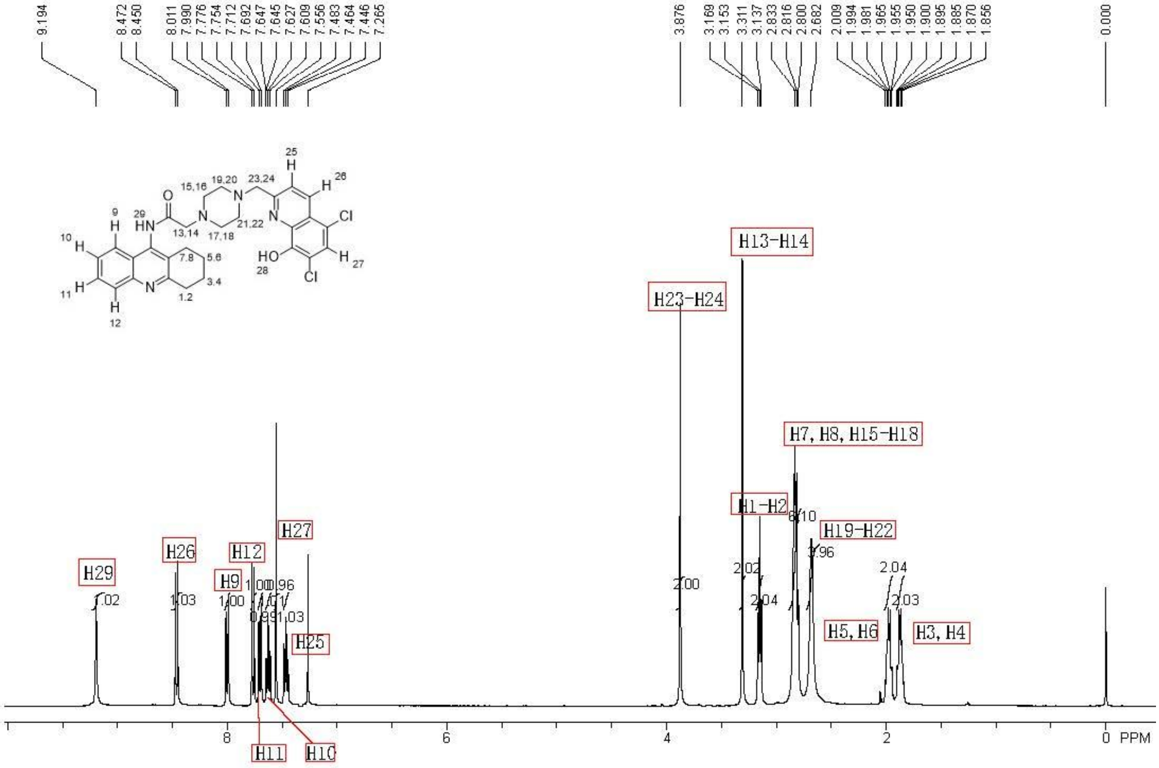

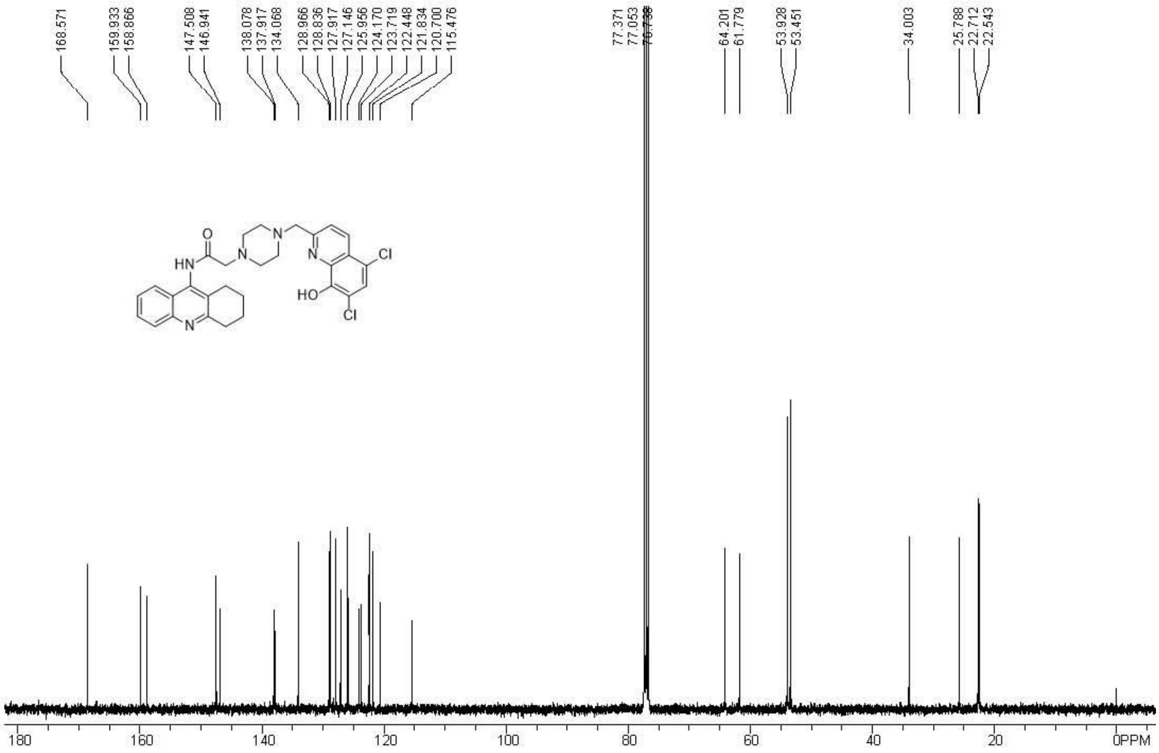

*2-(4-((8-Aminoquinolin-2-yl)methyl)piperazin-1-yl)-N-(1,2,3,4-tetrahydroacridin-9-yl)acetamide (**15f**)*

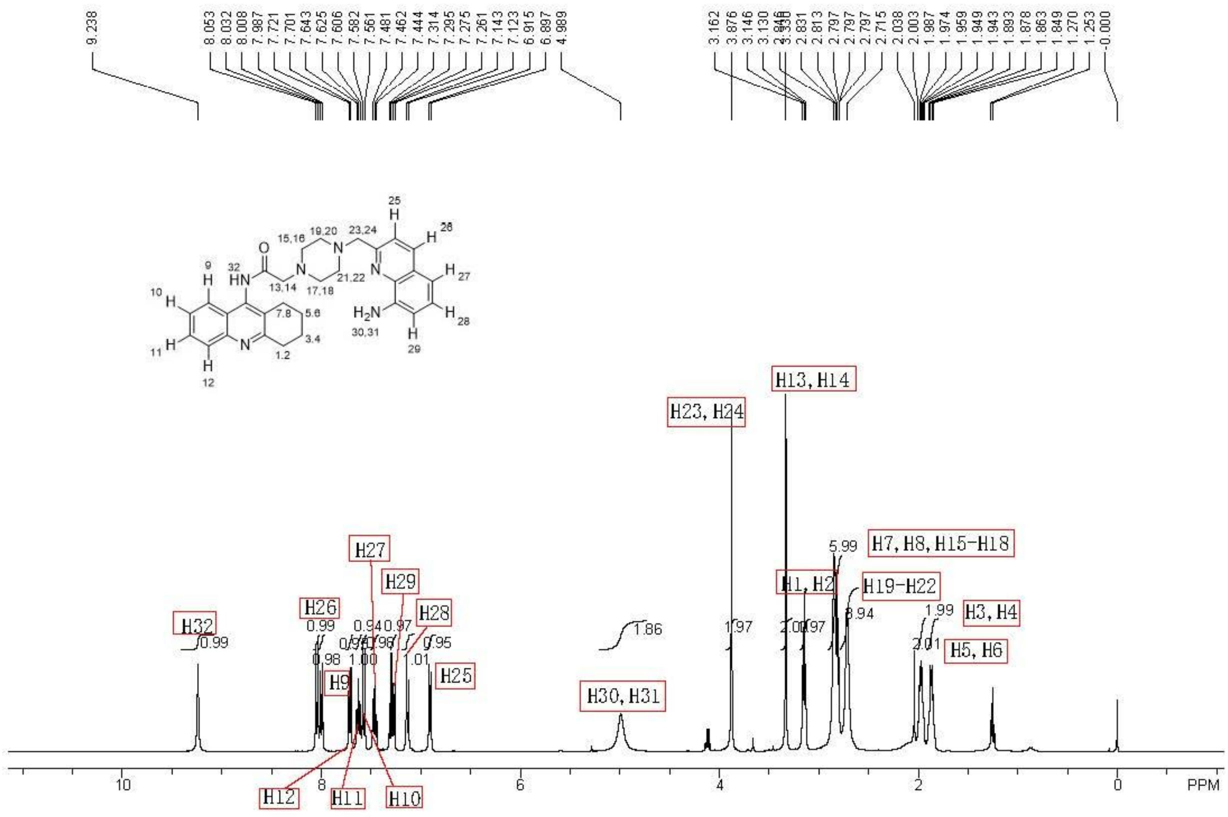

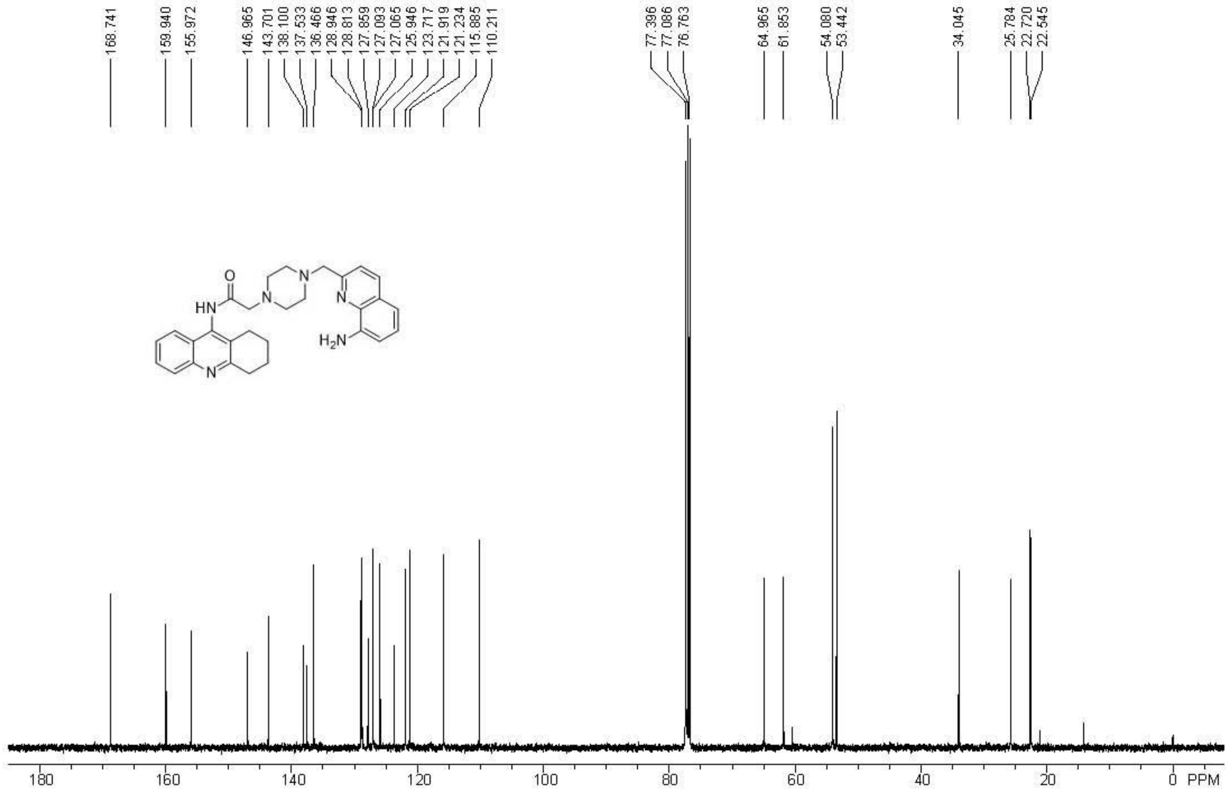

*2-((4-(2-((1,2,3,4-Tetrahydroacridin-9-yl)amino)ethyl)piperazin-1-yl)methyl)quinolin-8-ol (**16a**)*

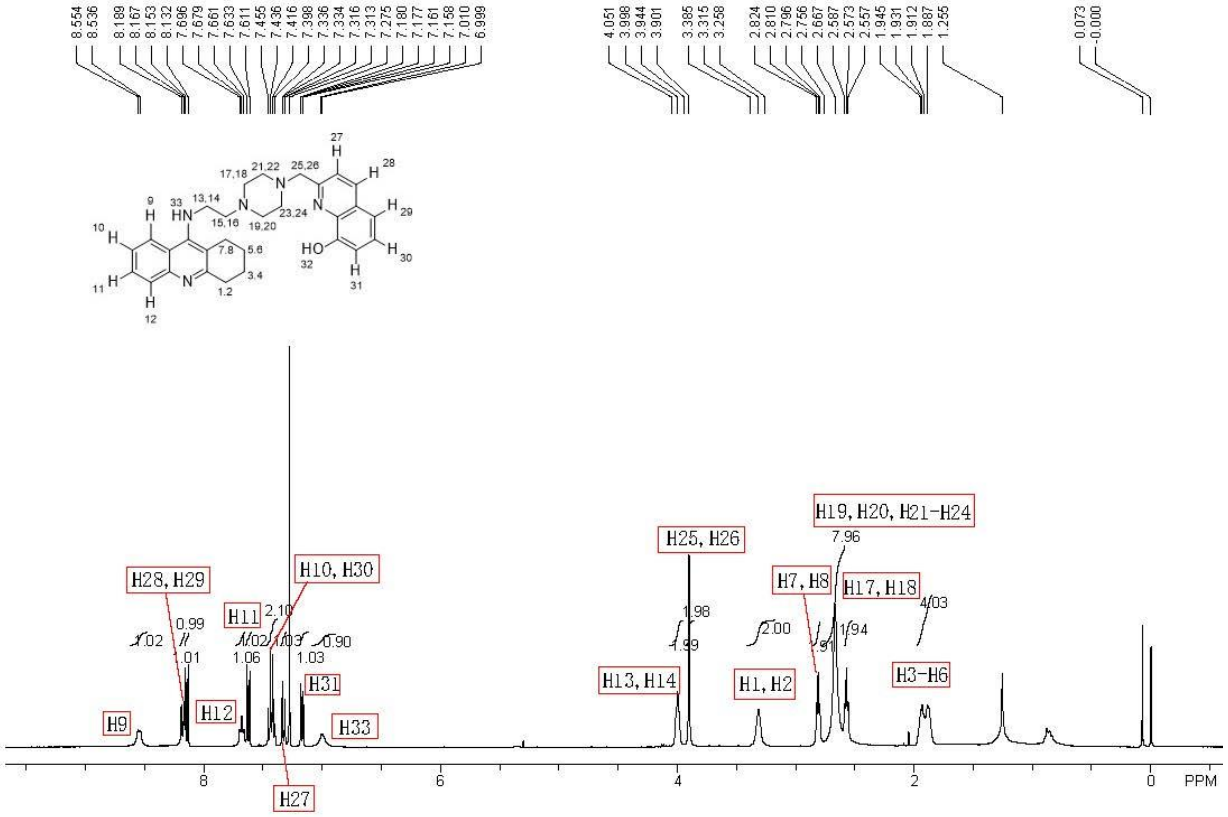

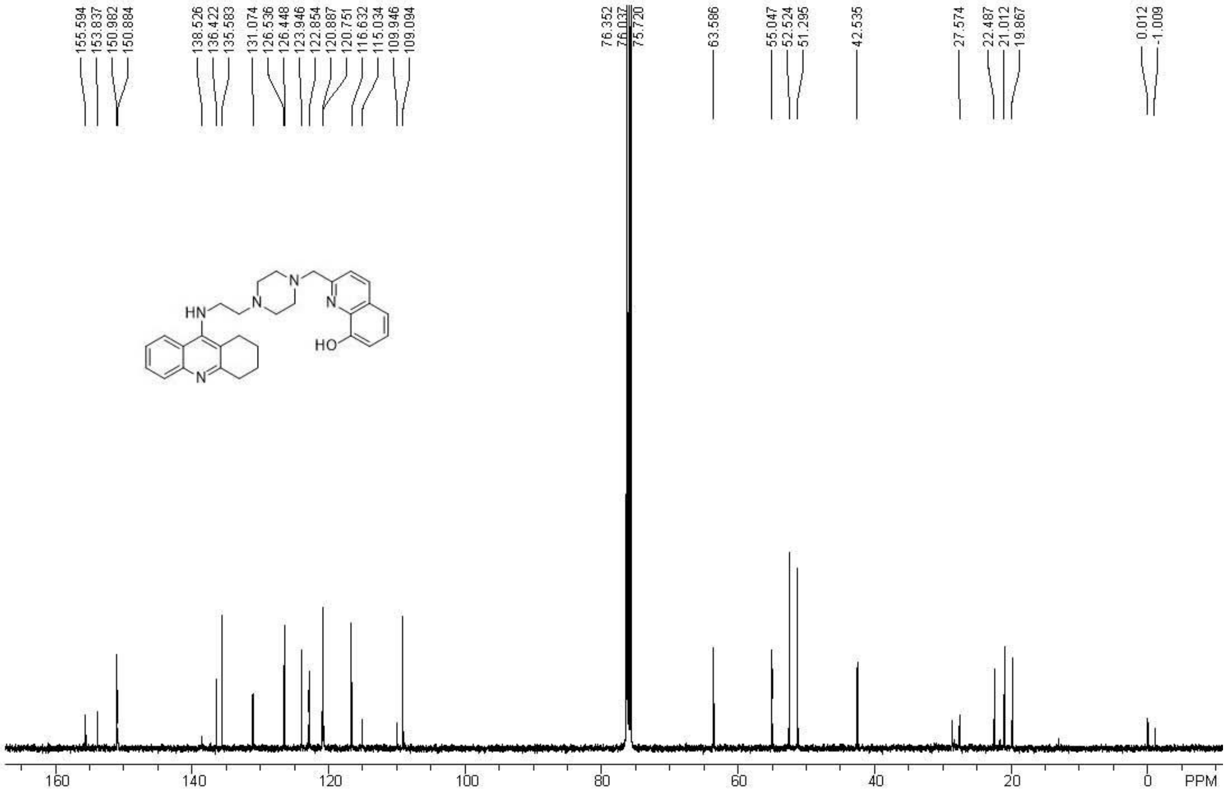

*5-Fluoro-2-((4-(2-((1,2,3,4-tetrahydroacridin-9-yl)amino)ethyl)piperazin-1-yl)methyl)quinolin-8-ol (**16b**)*

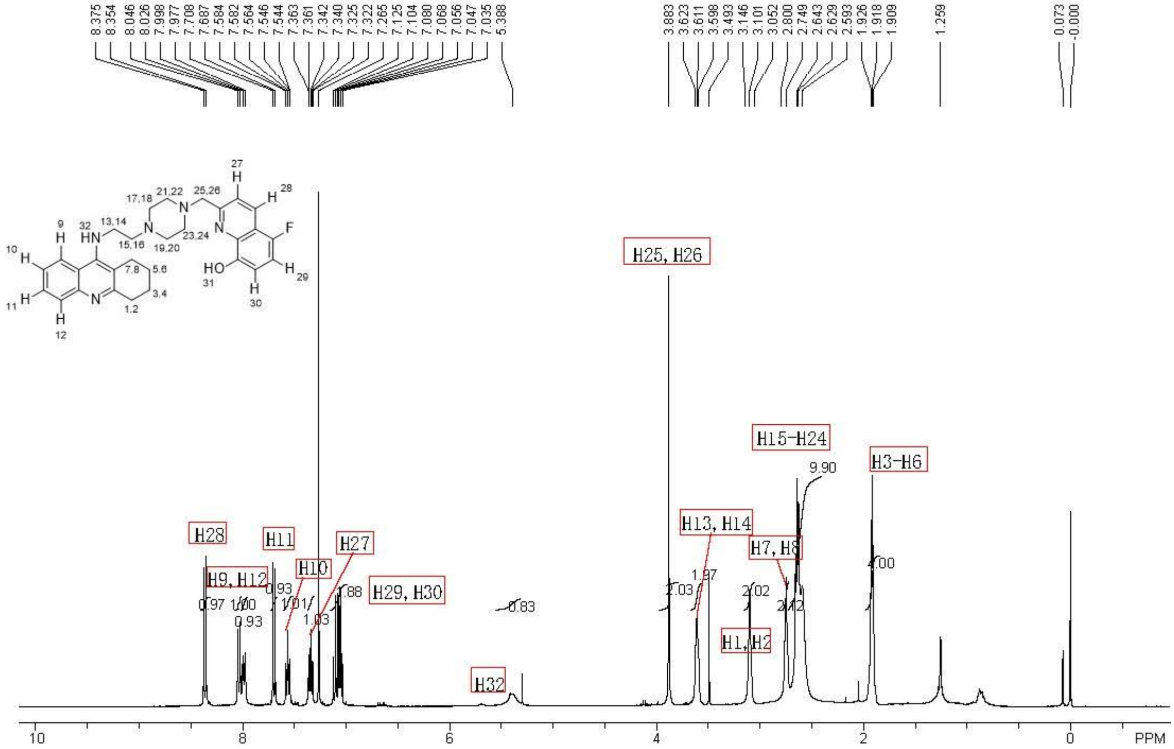

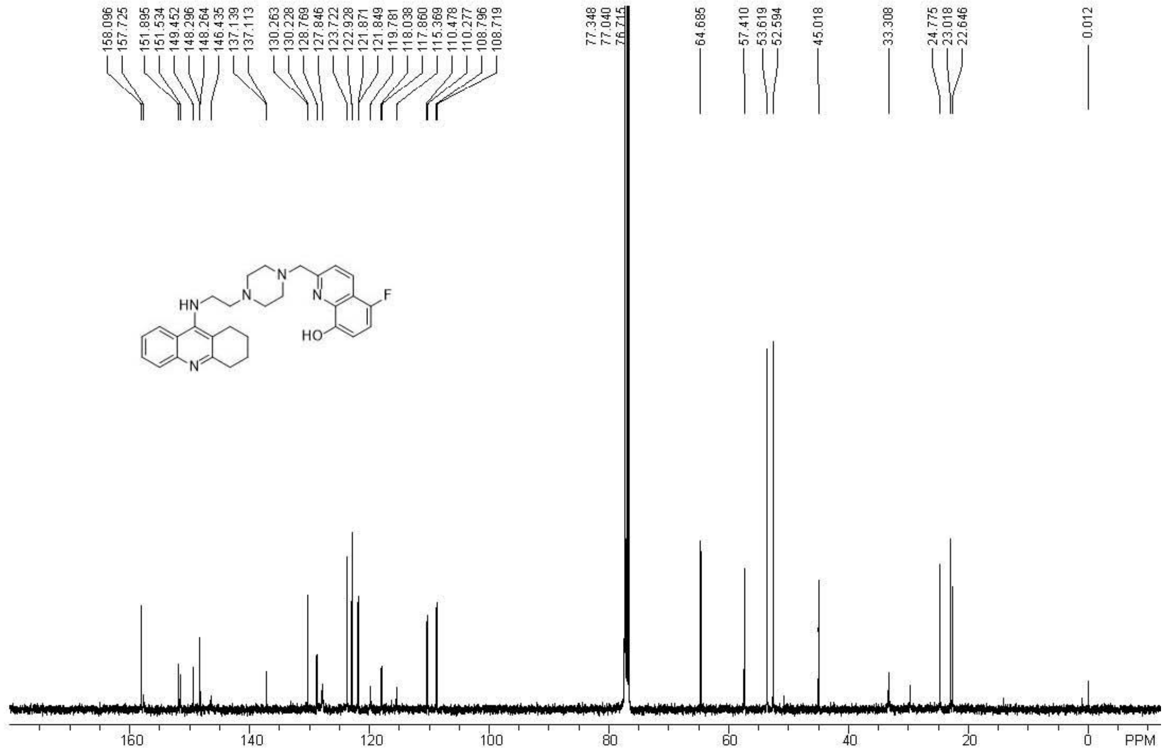

*5,7-Dichloro-2-((4-(2-((1,2,3,4-tetrahydroacridin-9-yl)amino)ethyl)piperazin-1-yl)methyl)quinolin-8-ol (**16c**)*

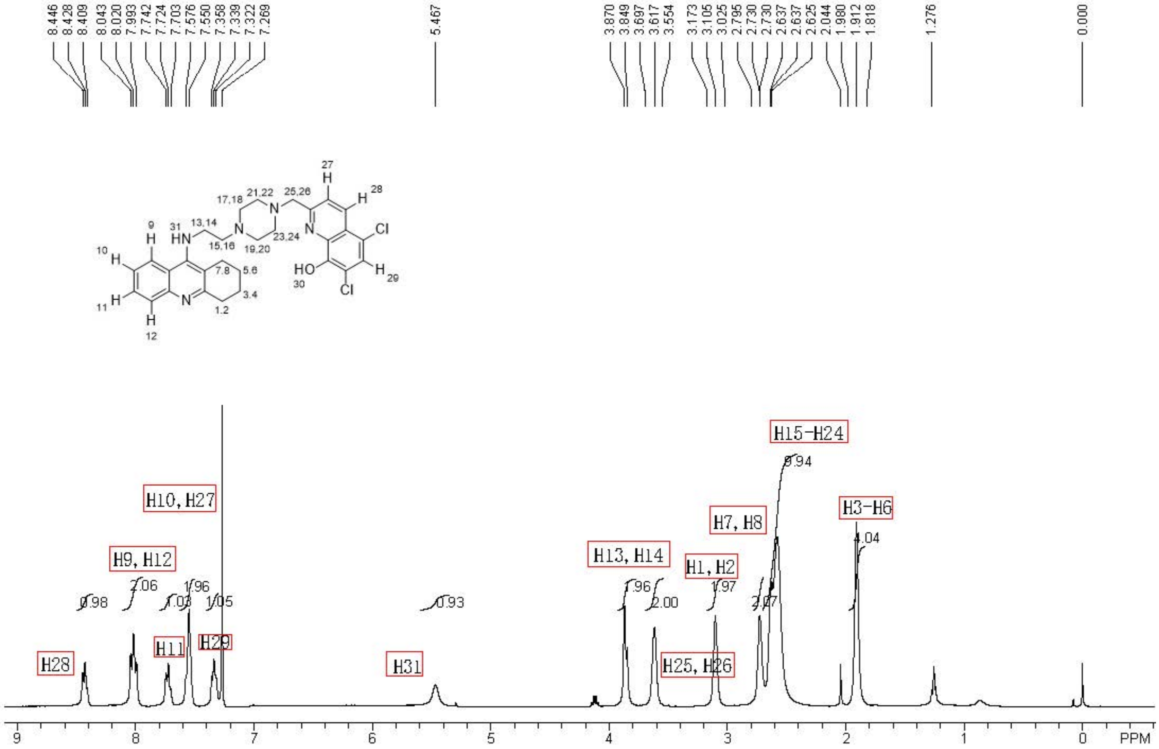

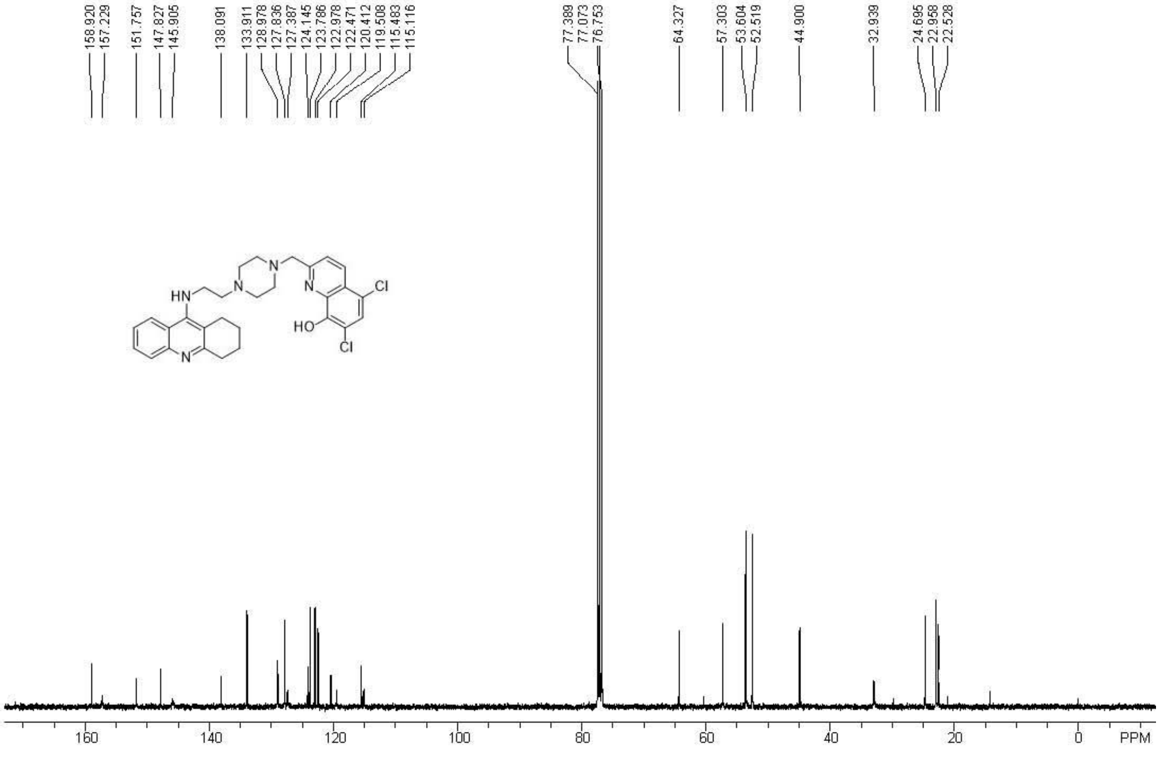

*N-(2-(4-((8-aminoquinolin-2-yl)methyl)piperazin-1-yl)ethyl)-1,2,3,4-tetrahydroacridin-9-amine (**16d**)*

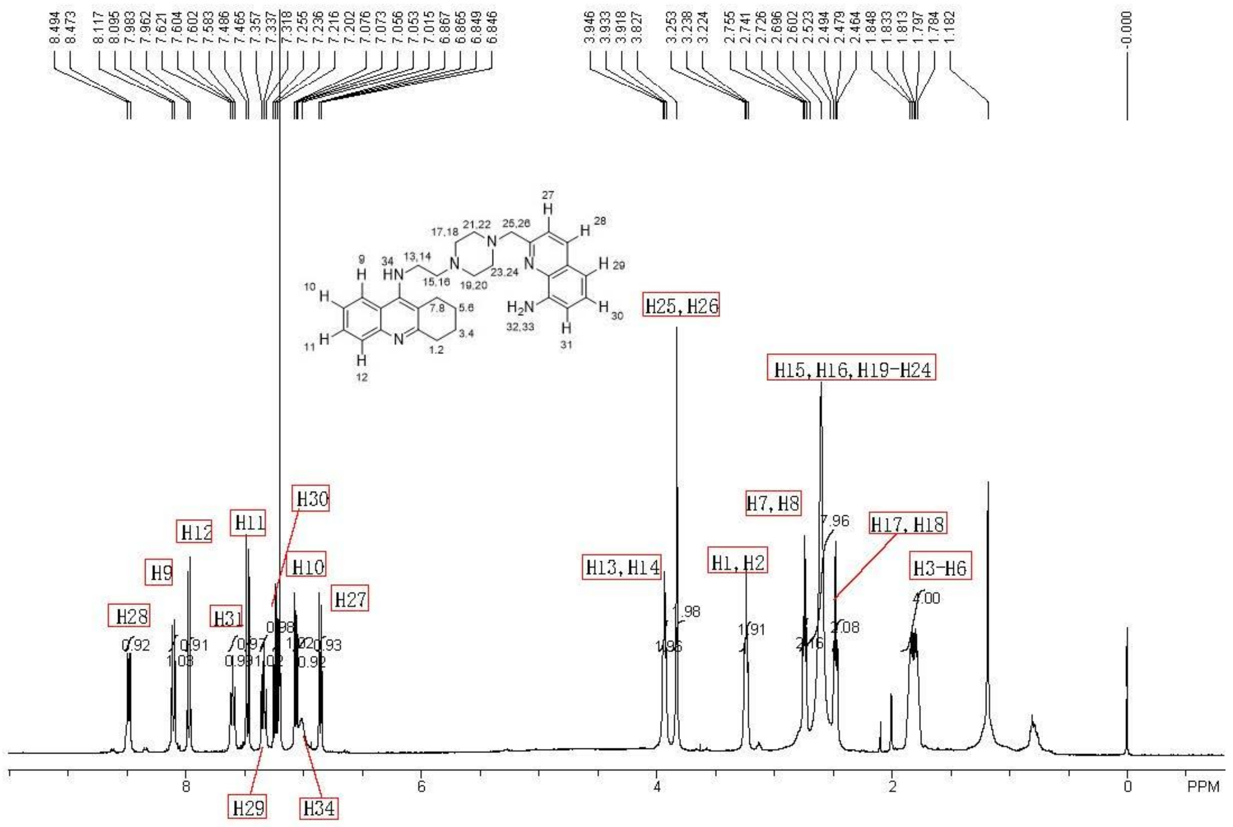

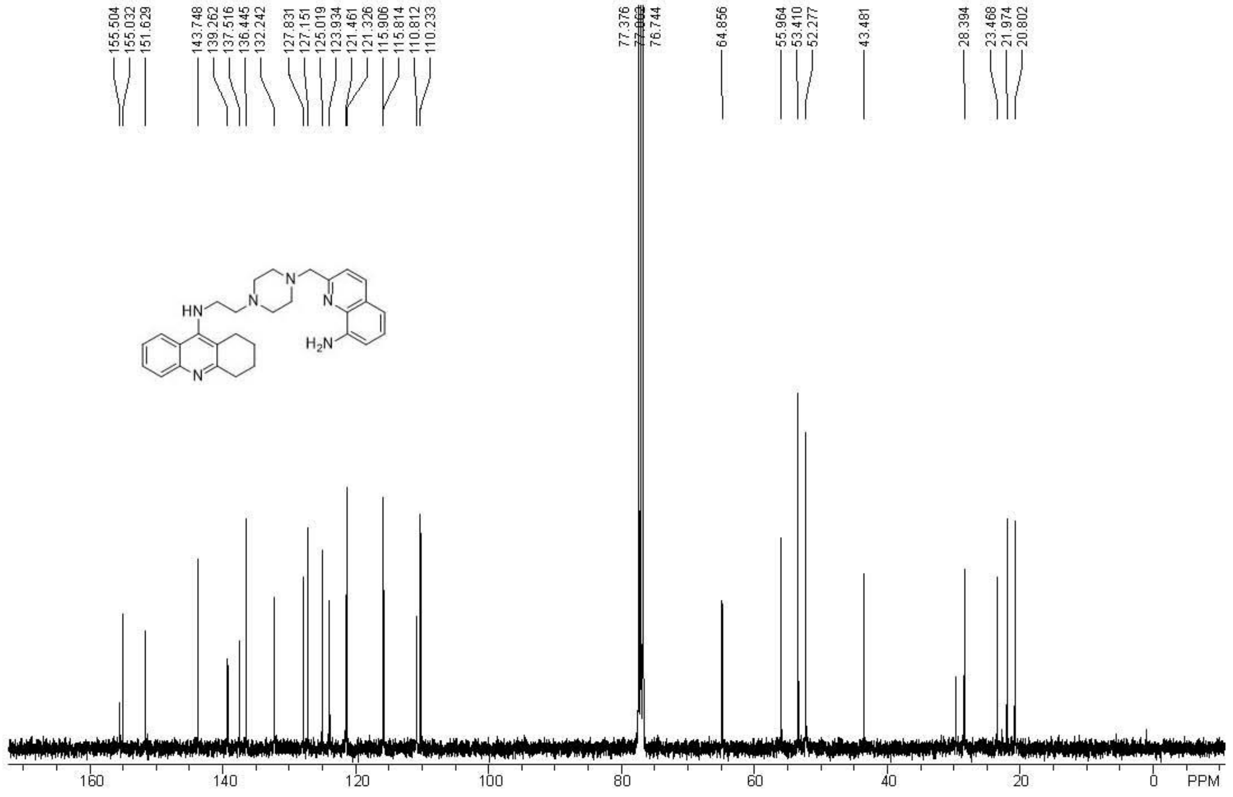

*2-((4-(3-((1,2,3,4-Tetrahydroacridin-9-yl)amino)propyl)piperazin-1-yl)methyl)quinolin-8-ol (**16e**)*

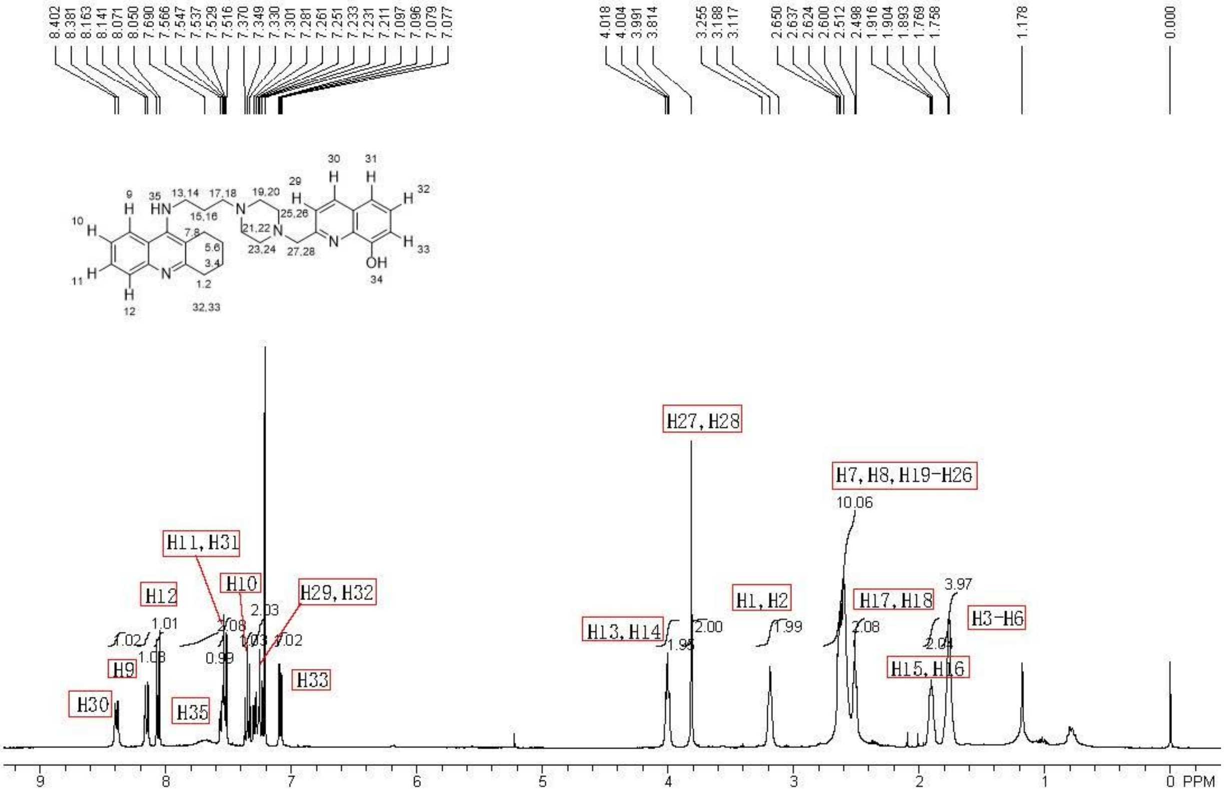

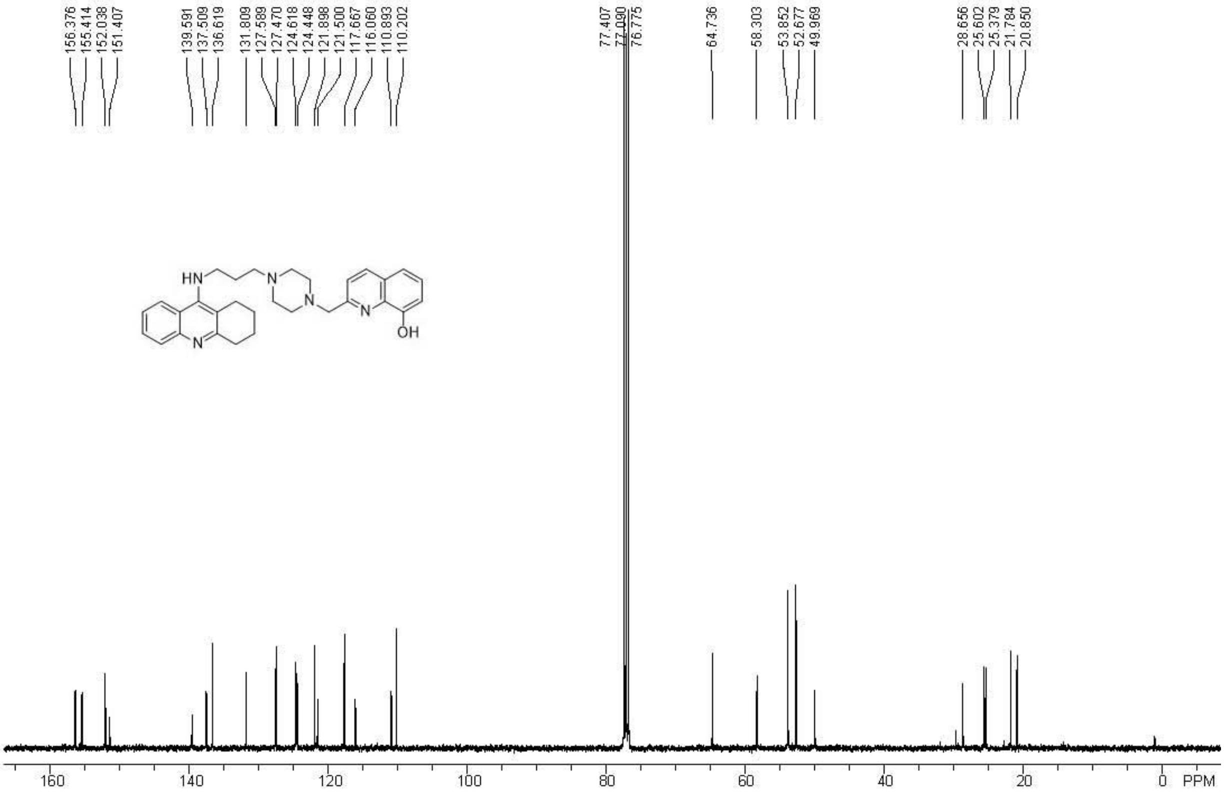

*5-Fluoro-2-((4-(3-((1,2,3,4-tetrahydroacridin-9-yl)amino)propyl)piperazin-1-yl)methyl)quinolin-8-ol (**16f**)*

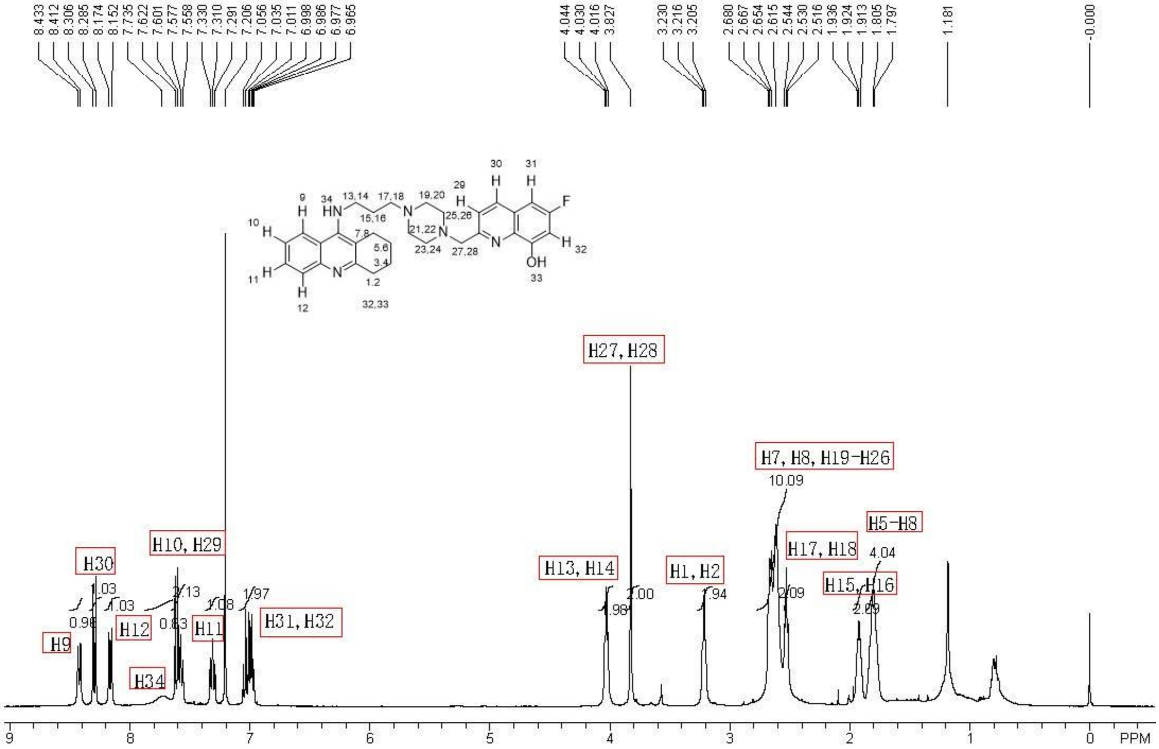

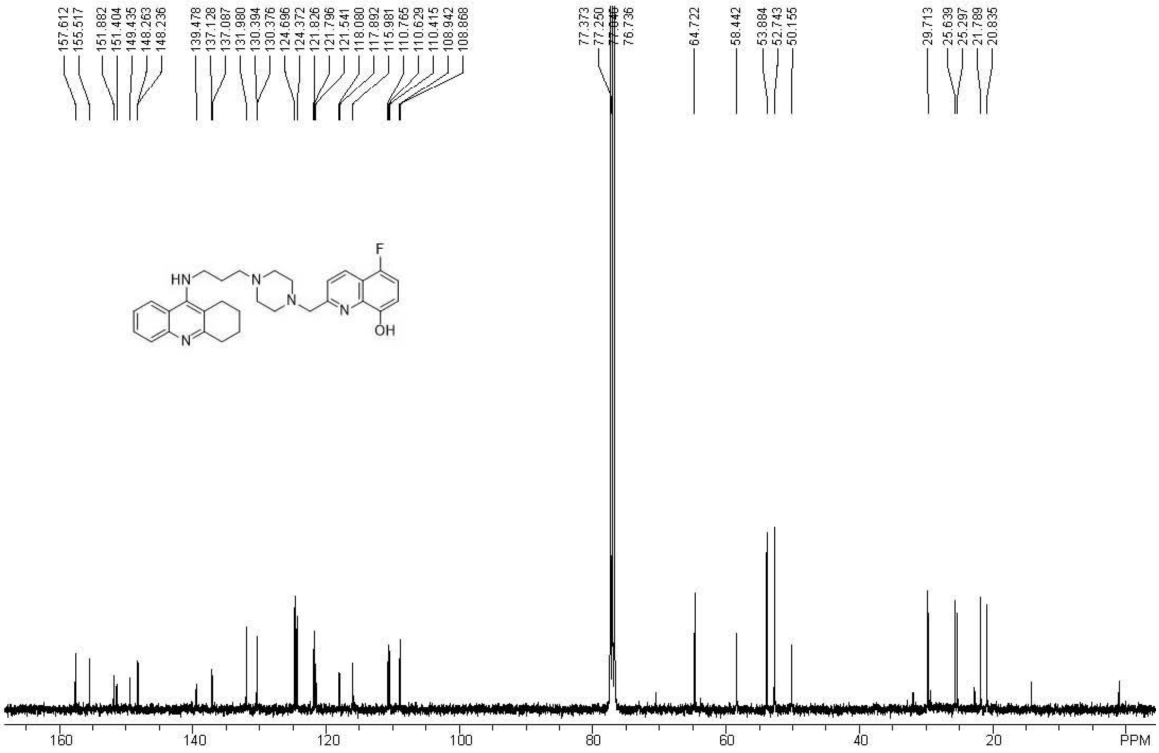

*N-(3-(4-((8-aminoquinolin-2-yl)methyl)piperazin-1-yl)propyl)-1,2,3,4-tetrahydroacridin-9-amine (**16g**)*

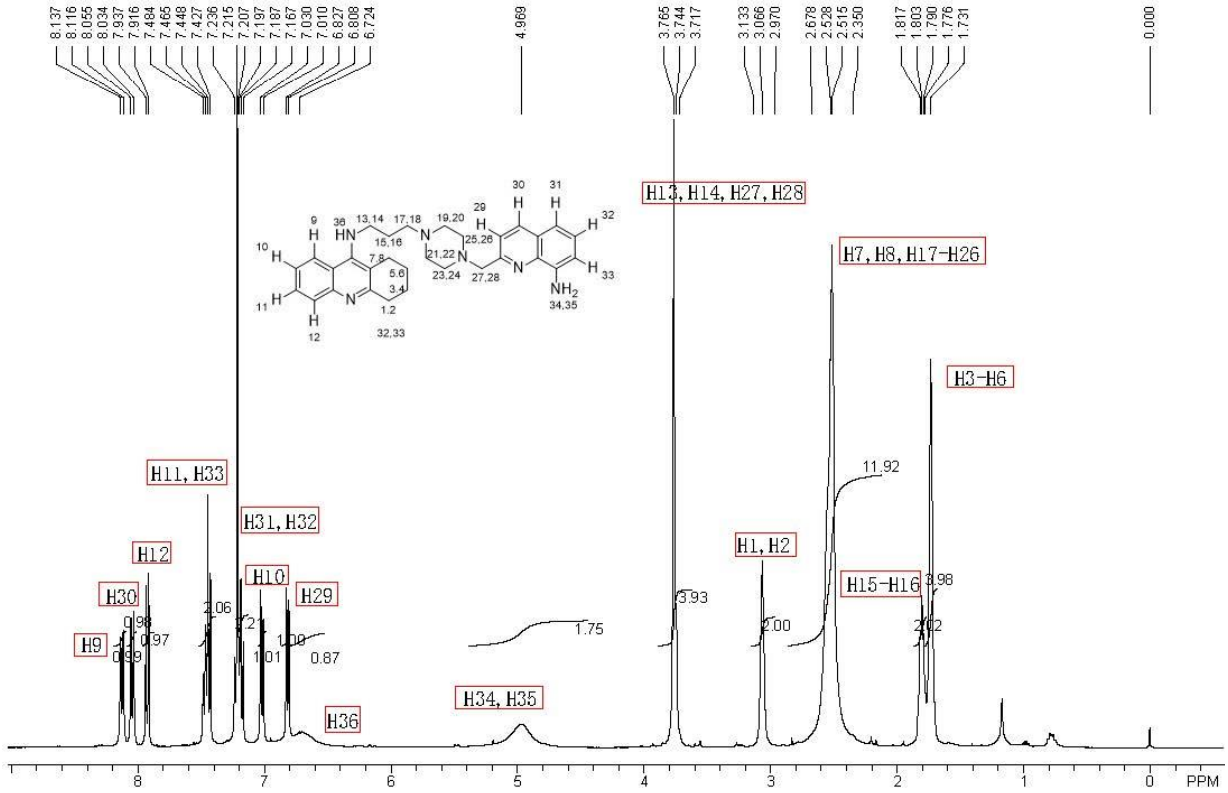

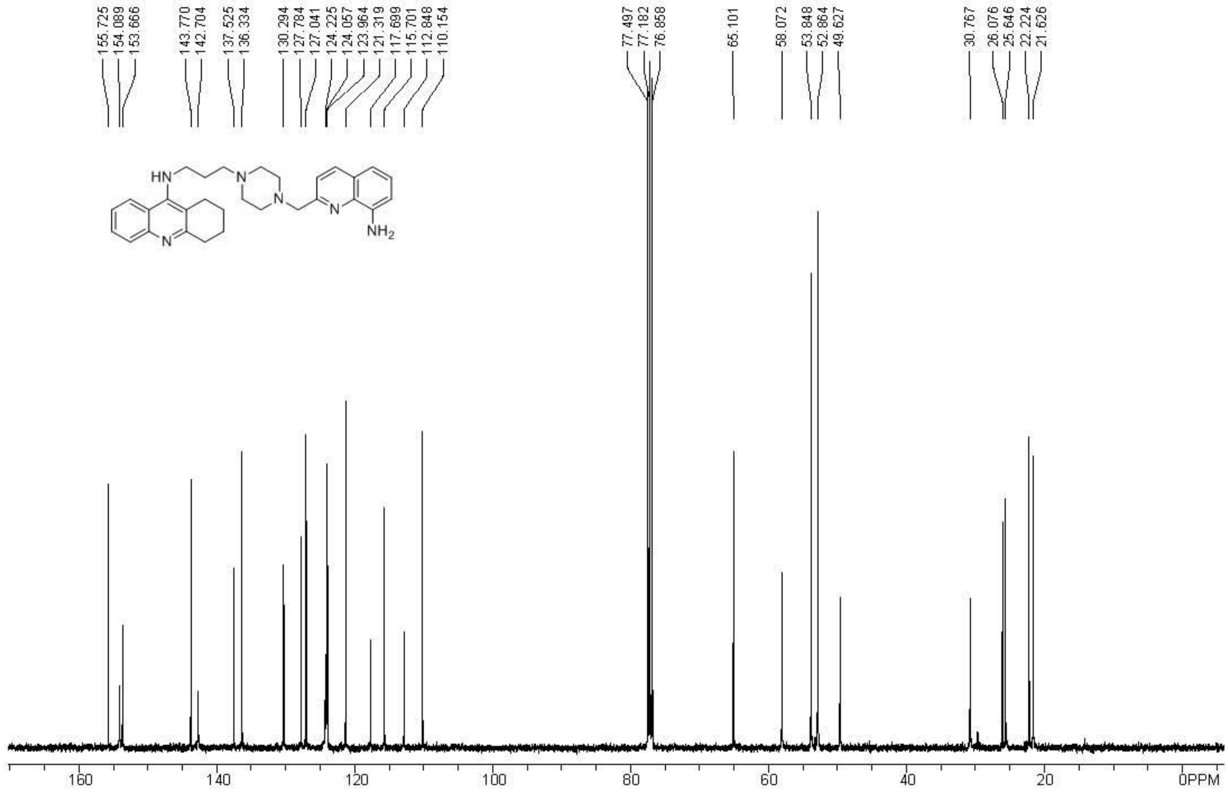

*2-((4-(5-((1,2,3,4-Tetrahydroacridin-9-yl)amino)pentyl)piperazin-1-yl)methyl)quinolin-8-ol (**16h**)*

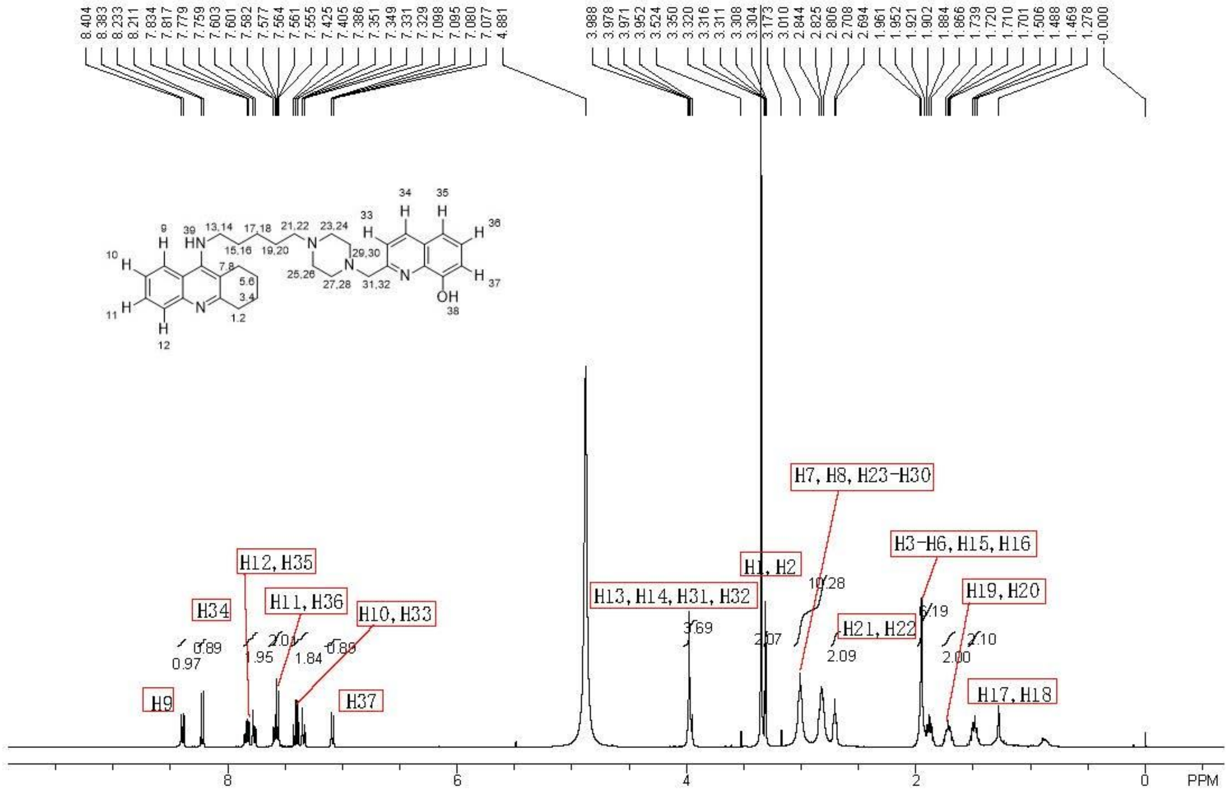

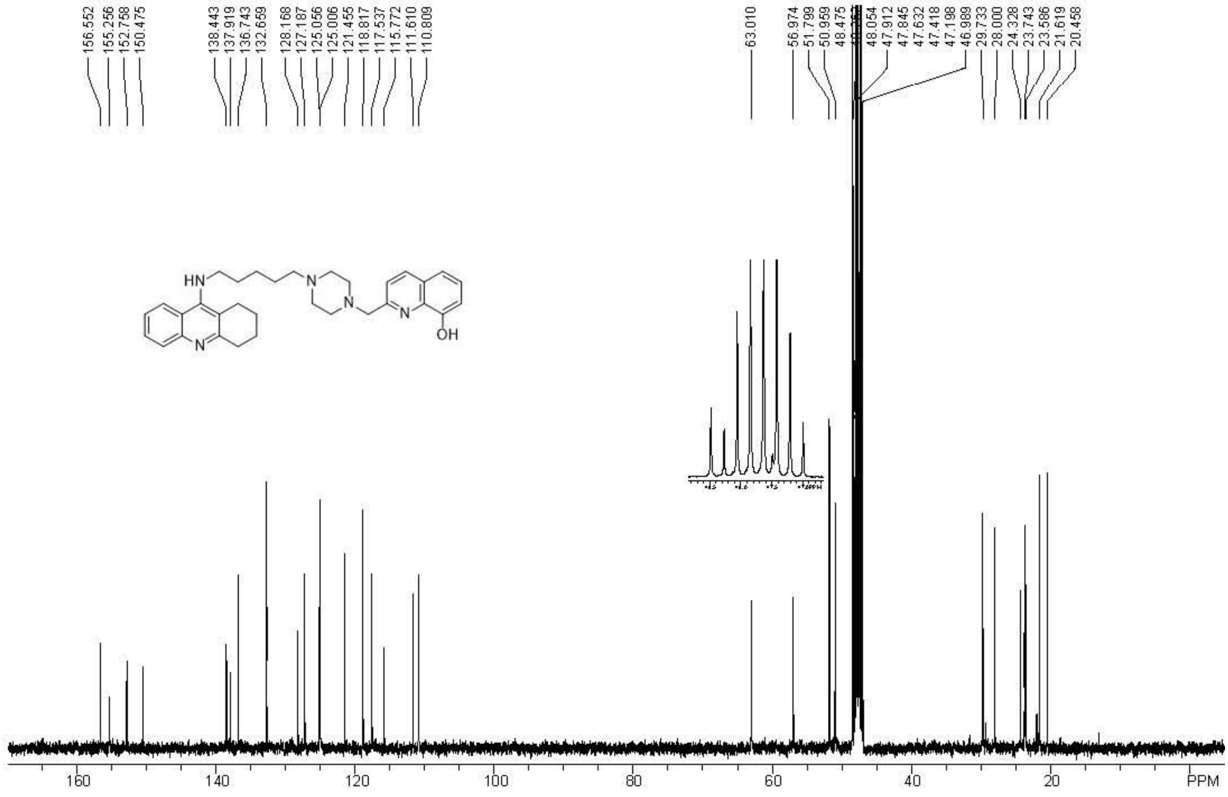

*5-Fluoro-2-((4-(5-((1,2,3,4-tetrahydroacridin-9-yl)amino)pentyl)piperazin-1-yl)methyl)quinolin-8-ol (**16i**)*

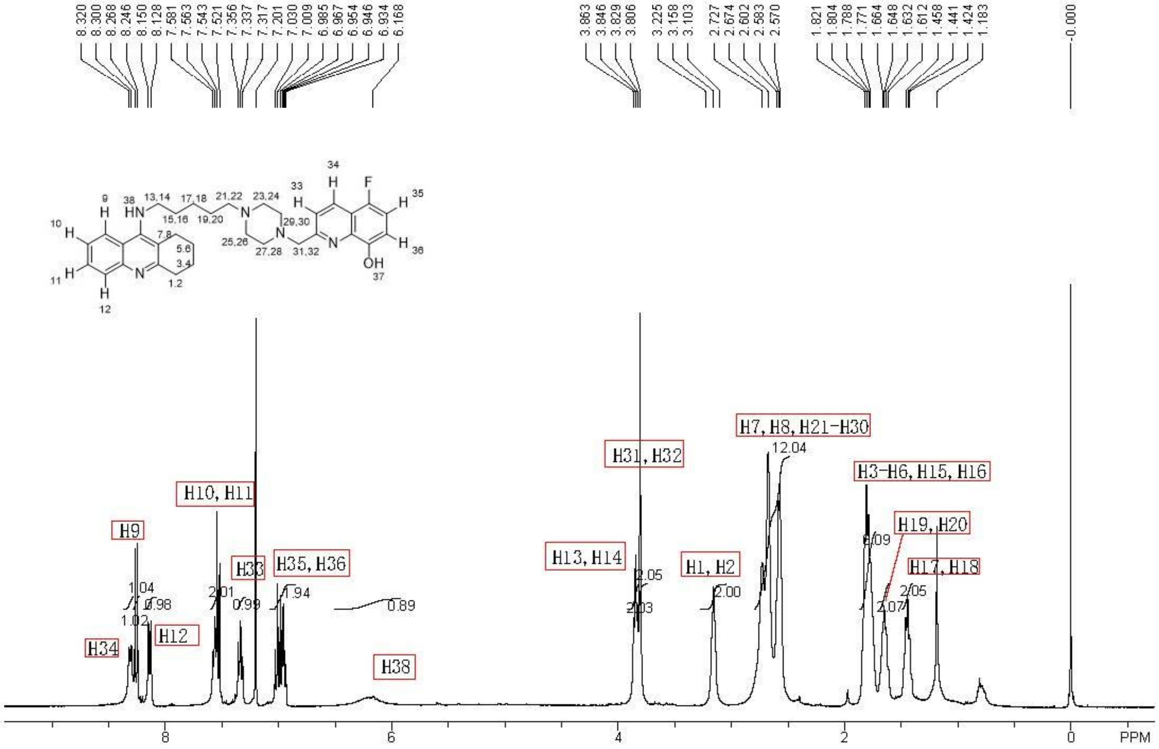

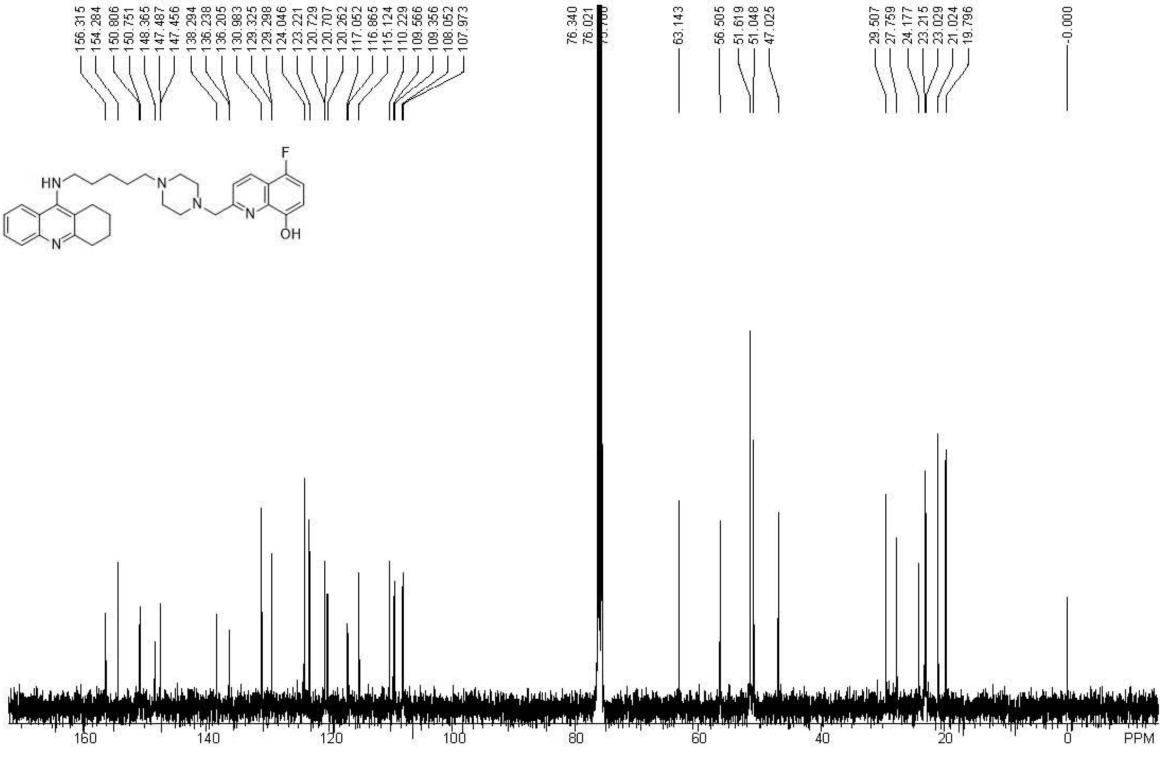

5-Chloro-2-((4-(5-((1,2,3,4-tetrahydroacridin-9-yl)amino)pentyl)piperazin-1-yl)methyl)quinolin-8-ol ***(16j)***

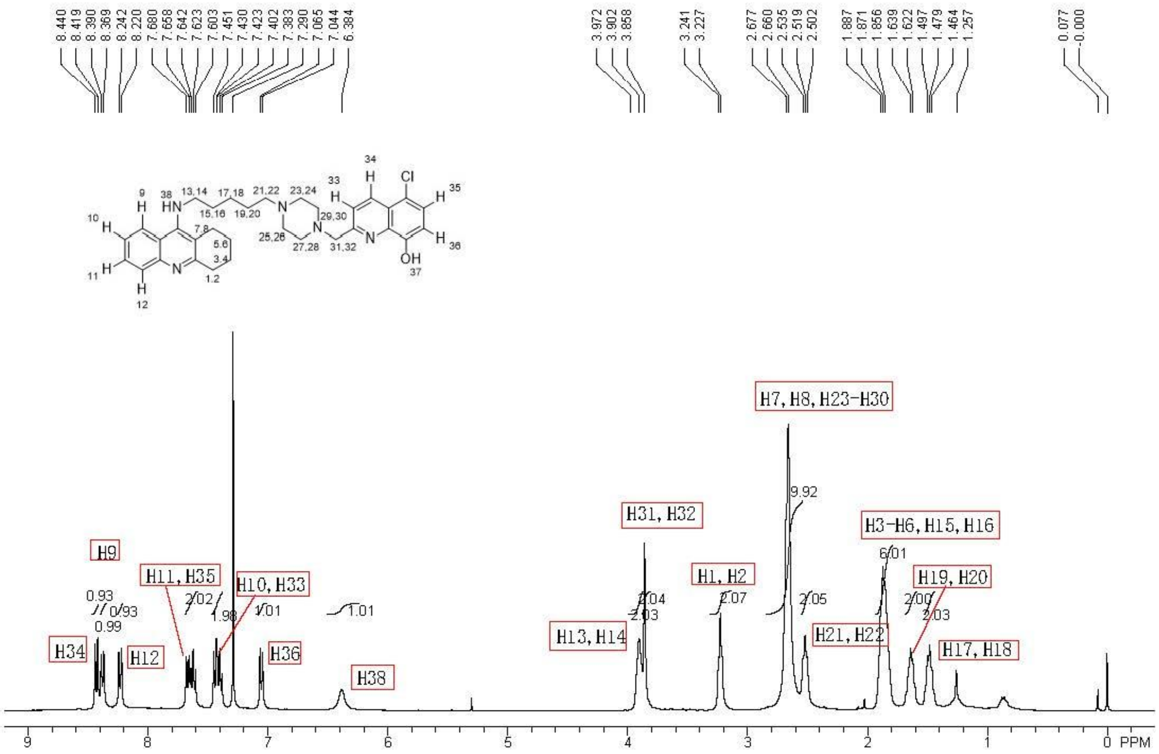

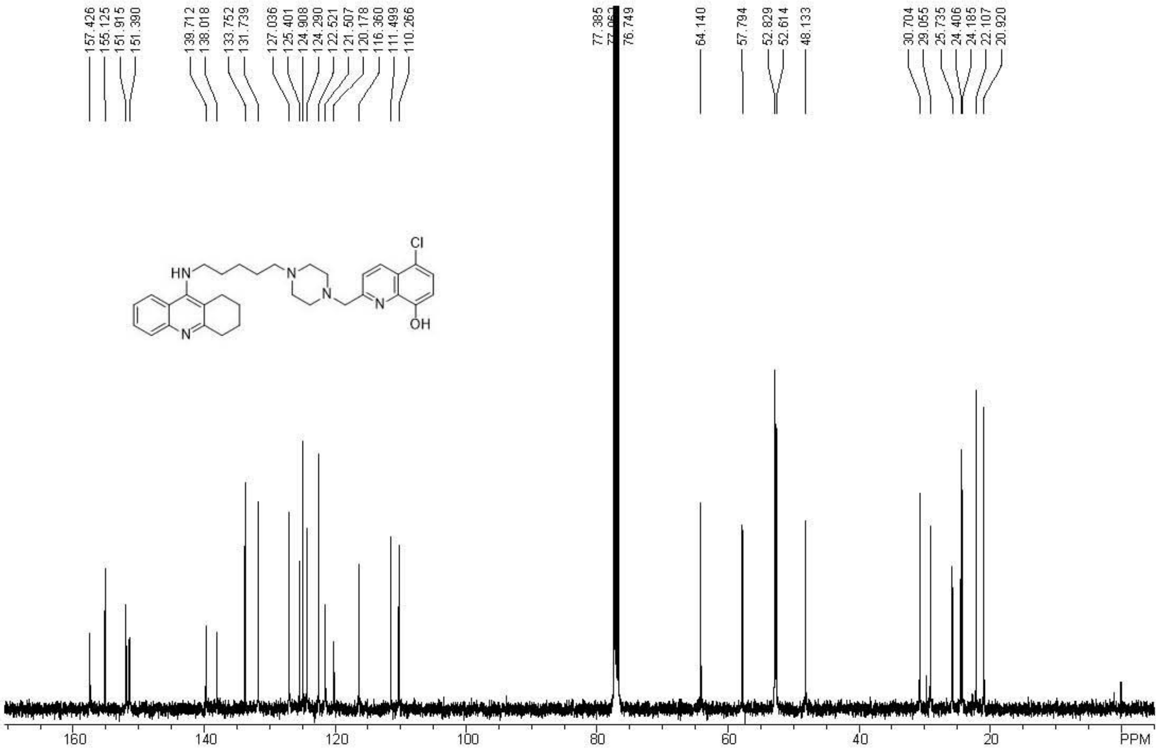

*N-(5-(4-((8-aminoquinolin-2-yl)methyl)piperazin-1-yl)pentyl)-1,2,3,4-tetrahydroacridin-9-amine (**16k**)*

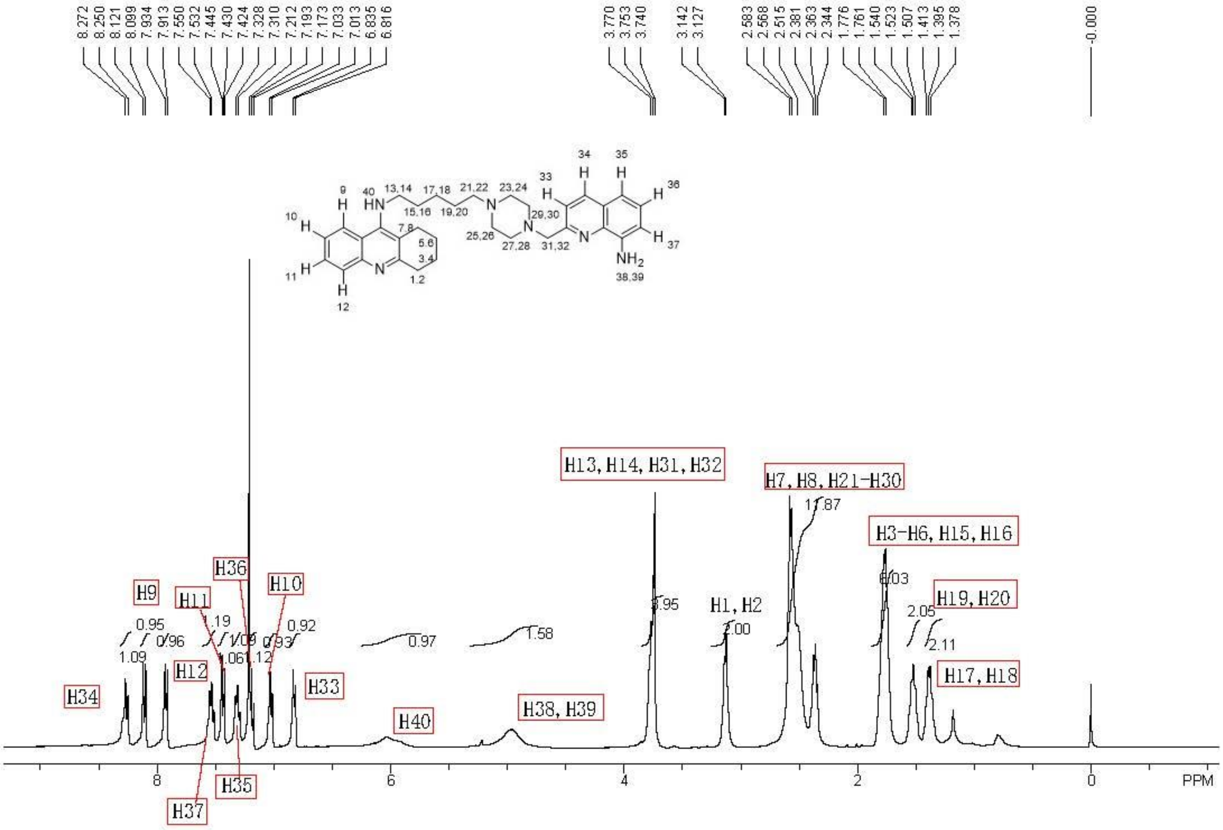

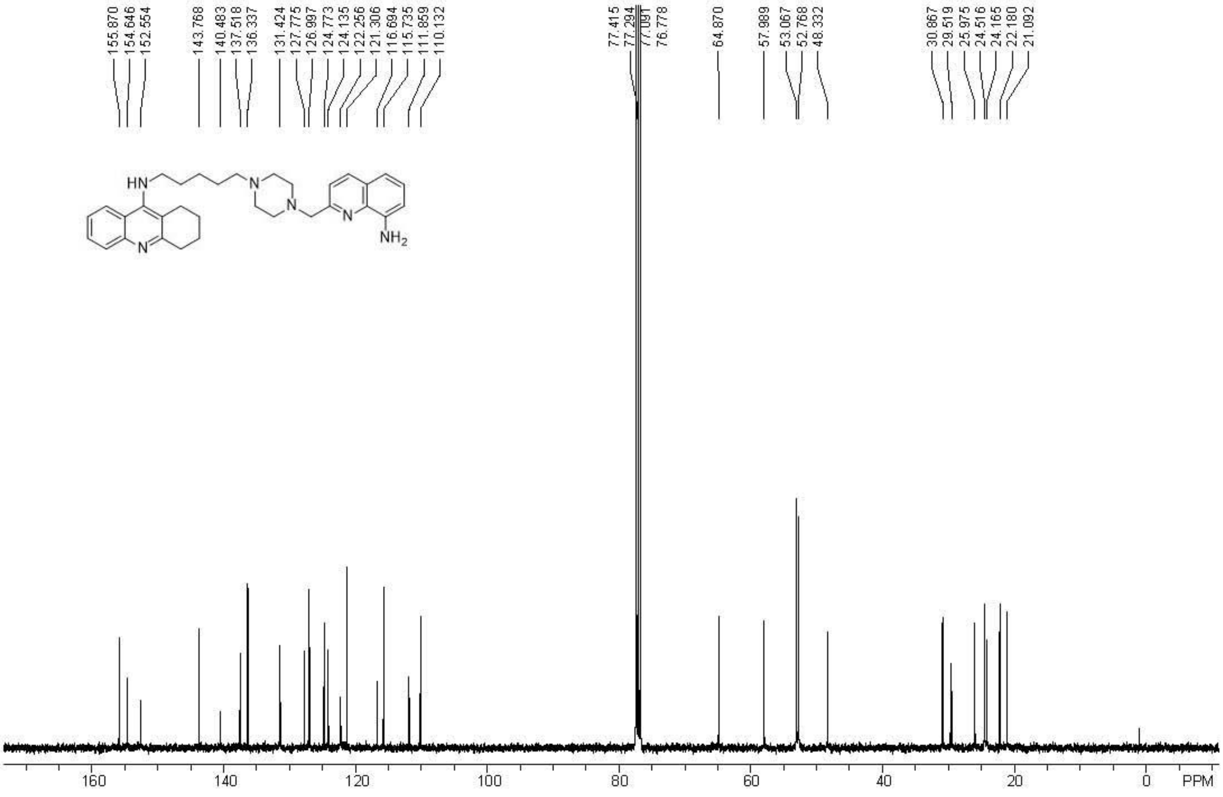

*2-(4-((8-Hydroxyquinolin-5-yl)methyl)piperazin-1-yl)-N-(1,2,3,4-tetrahydroacridin-9-yl)acetamide (**17a**)*

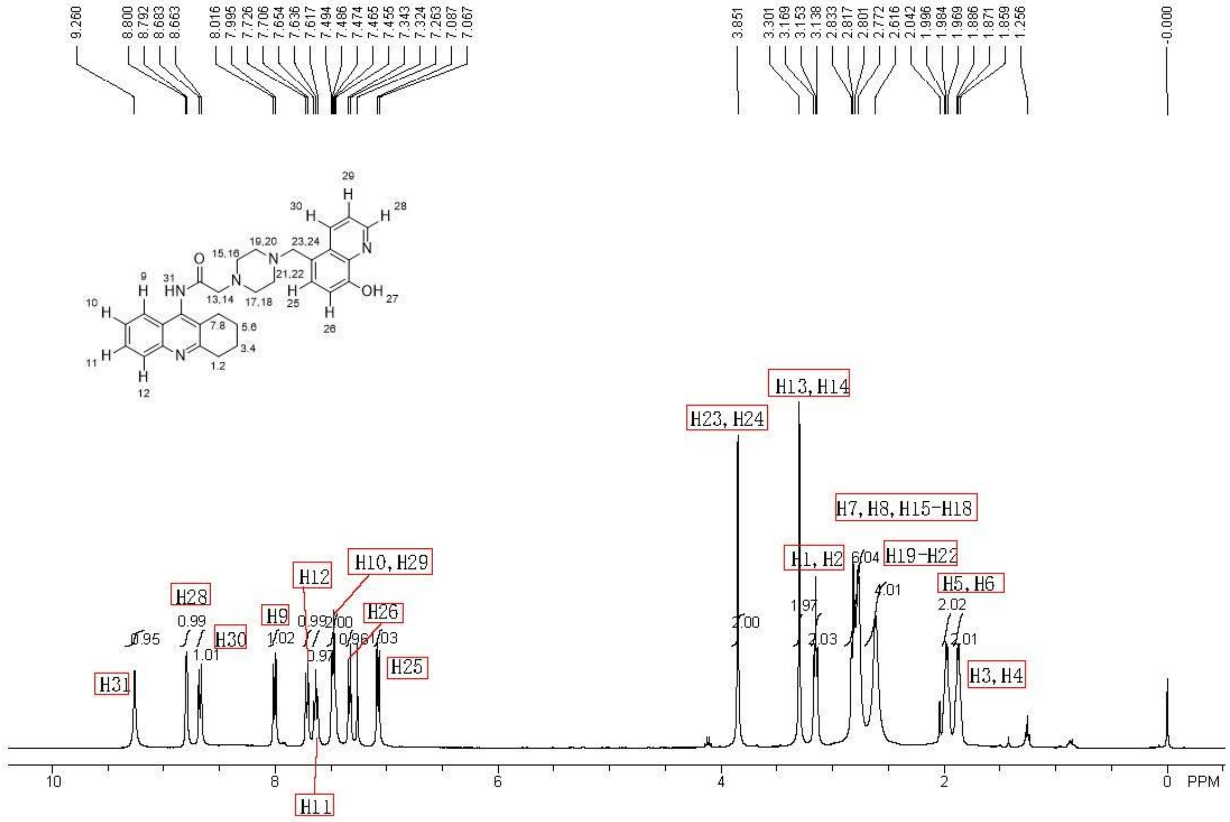

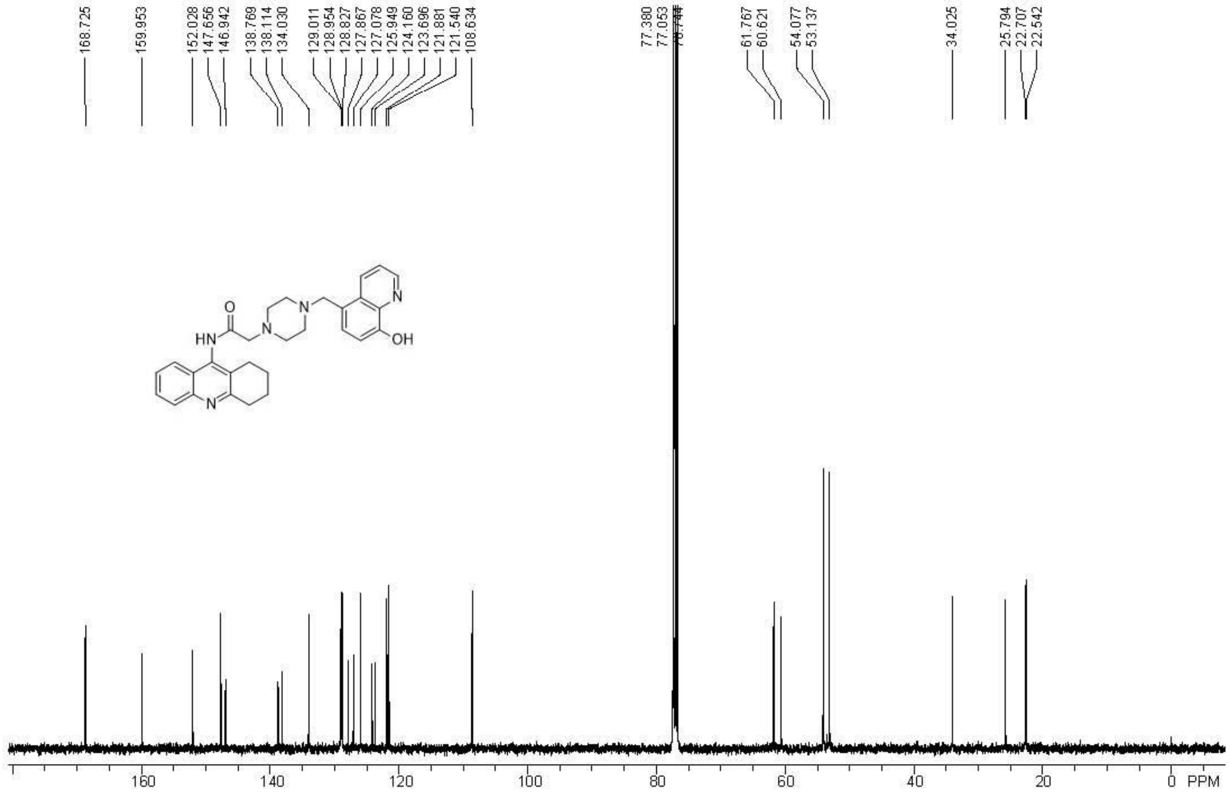

*2-(4-((8-Hydroxy-2-methylquinolin-5-yl)methyl)piperazin-1-yl)-N-(1,2,3,4-tetrahydroacridin-9-yl)acetamide (**17b**)*

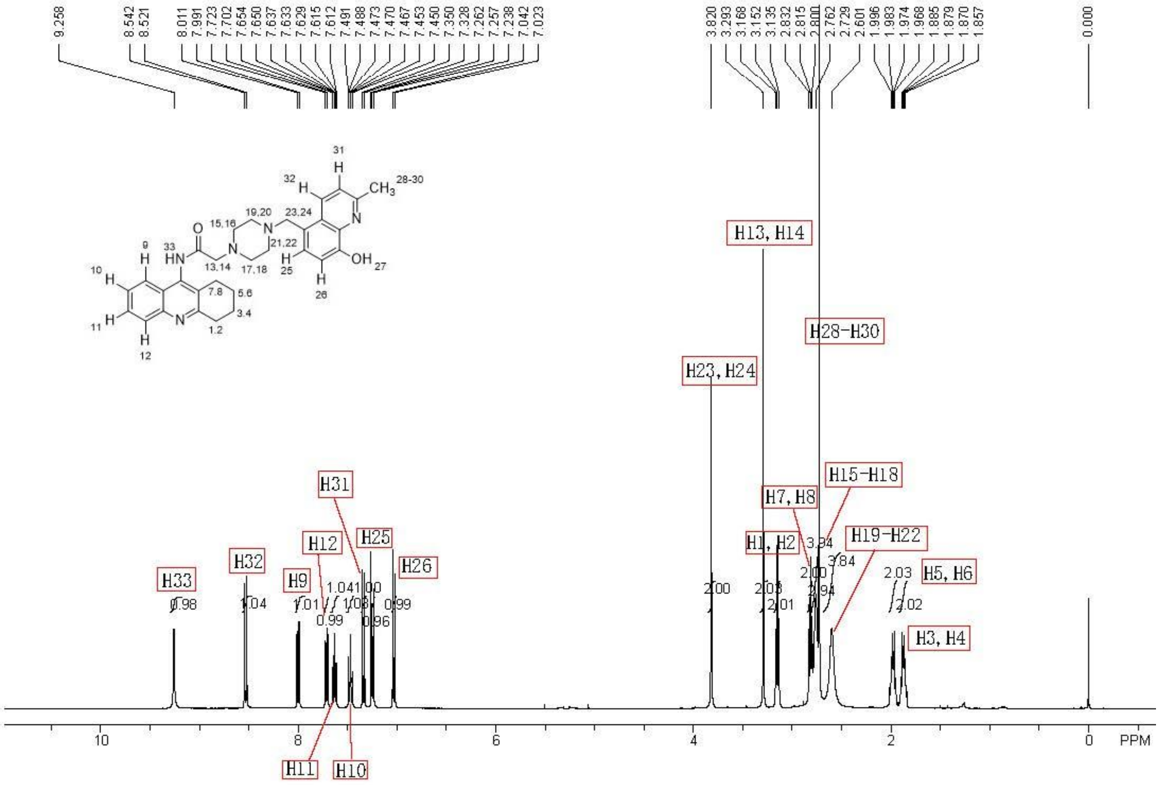

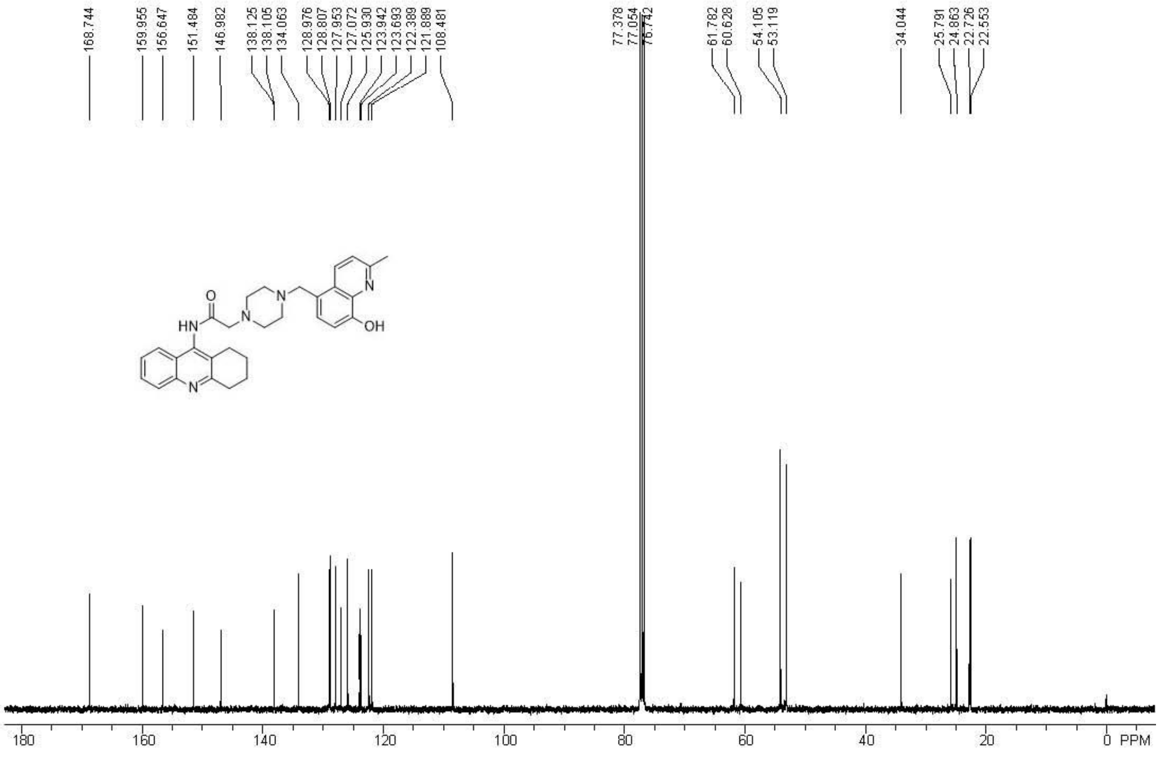

Molecular Docking

A structure–activity relationship (SAR) comparison highlighted that 8-aminoquinoline derivatives (e.g., 15f, 16d, 16g, and 16k) generally exhibited stronger inhibitory activities against AChE and Aβ aggregation; however, they lacked reliable metal-chelating ability. In contrast, 8-hydroxyquinoline derivatives displayed a more balanced multitarget profile, combining potent cholinesterase inhibition, effective suppression of Aβ aggregation, and robust metal-chelating properties. Docking studies further supported the SAR findings. Compound **15a** mainly interacted with residues at the peripheral site of BuChE (e.g., PRO-285, SER-287), whereas **17a** extended deeper into the catalytic gorge and formed hydrogen bonds with residues near the catalytic triad (GLU-197, SER-198). This dual-site engagement of **17a** is consistent with its stronger BuChE inhibitory activity compared with the 15 series

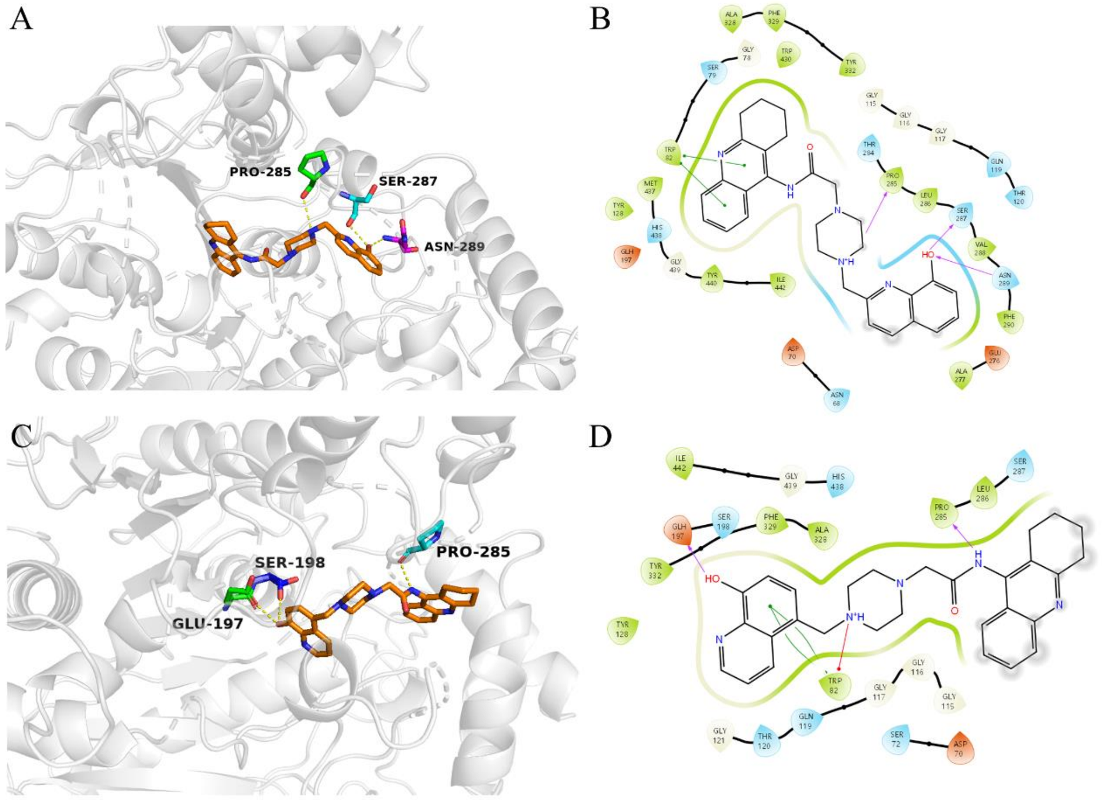

Figure S1. Molecular docking poses of compounds **15a** (A, B) and **17a** (C, D) with BuChE (PDB ID: 1POI).
